# Effect of metformin on left ventricular mass and functional parameters in non-diabetic patients: a meta-analysis of randomized clinical trials

**DOI:** 10.1186/s12872-022-02845-w

**Published:** 2022-09-10

**Authors:** Ahmed M. Kamel, Nirmeen Sabry, Samar Farid

**Affiliations:** Clinical Pharmacy Department, Faculty of Pharmacy, Cairo University, Cairo, 11562 Egypt

**Keywords:** Left ventricular mass index, Metformin, Left ventricular ejection fraction, Cardiovascular disease, Systematic review, Meta-analysis

## Abstract

**Background:**

Left ventricular hypertrophy is a common finding in patients with ischemic heart disease and is associated with mortality in patients with cardiovascular disease (CVD). Metformin, an antidiabetic drug, has been shown to reduce oxidative stress and left ventricular mass index (LVMI) in animal hypertrophy models. We summarized evidence regarding the effect of metformin on LVMI and LVEF.

**Methods:**

Electronic databases were searched for randomized clinical trials (RCTs) that used metformin in non-diabetic patients with or without pre-existing CVD. The standardized mean change using change score standardization (SMCC) was calculated for each study. The random-effects model was used to pool the SMCC across studies. Meta-regression analysis was used to assess the association of heart failure (HF), metformin dose, and duration with the SMCC.

**Results:**

Data synthesis from nine RCTs (754 patients) showed that metformin use resulted in higher reduction in LVMI after 12 months (SMCC = −0.63, 95% CI − 1.23; − 0.04, p = 0.04) and an overall higher reduction in LVMI (SMCC = −0.5, 95% CI − 0.84; − 0.16, p < 0.01). These values equate to absolute values of 11.3 (95% CI 22.1–0.72) and 8.97 (95% CI 15.06–2.87) g/m^2^, respectively. The overall improvement in LVEF was also higher in metformin users after excluding one outlier (SMCC = 0.26, 95% CI 0.03–0.49, P = 0.03) which translates to a higher absolute improvement of 2.99% (95% CI 0.34; 5.63). Subgroup analysis revealed a favorable effect for metformin on LVEF in patients who received > 1000 mg/day (SMCC = 0.28, 95% CI 0.04; 0.52, P = 0.04), and patients with HF (SMCC = 0.23; 95% CI 0.1; 0.36; P = 0.004). These values translate to a higher increase of 2.64% and 3.21%, respectively.

**Conclusion:**

Results suggest a favorable effect for metformin on LVMI and LVEF in patients with or without pre-existing CVD. Additional trials are needed to address the long-term effect of metformin.

*Registration* The study was registered on the PROSPERO database with the registration number CRD42021239368 (https://www.crd.york.ac.uk/prospero).

**Supplementary Information:**

The online version contains supplementary material available at 10.1186/s12872-022-02845-w.

## Highlights

1. Metformin therapy regresses LVH by approximately 10 g/m^2^ in non-diabetic patients after 12 months of use (SMCC = −0.63, 95% CI − 1.23; − 0.04, p = 0.04).
2. Metformin therapy was associated with a modest (2–3%) but greater overall improvement in LVEF.
3. Higher dose (> 1000 mg/day) and longer treatment duration were associated with a significant effect for metformin on LVMI and LVEF.
4. Metformin use in HF patients was associated with an absolute increase of 3.21% in LVEF (SMCC = 0.23; 95% CI 0.1; 0.36, P = 0.004).

**Supplementary Information:**

The online version contains supplementary material available at 10.1186/s12872-022-02845-w.

## Introduction

Left ventricular hypertrophy (LVH) is a common finding in patients with ischemic heart disease and is associated with mortality even in the absence of hypertension [1]. LVH is also present in approximately one-third of coronary artery disease (CAD) patients [2]. Left ventricular mass index (LVMI), assessed using echocardiography and cardiac magnetic resonance imaging (CMR), can be used to assess structural heart disease in combination with measures of diastolic function [3, 4]. Prognostically, the presence of LVH is associated with worse prognosis and all-cause mortality in patients with stable CAD [5]. Moreover, the risk of all-cause mortality was four times higher in CAD patients with LVH than CAD patients without LVH while a relative risk of 2.14 (95% CI 1.24–3.68) was reported in patients without CAD [6]. Another study reported that concentric remodeling was associated with higher risk of stroke and CAD [7]. The risk of death or non-fatal complications was two to four-folds higher in the presence of LVH, irrespective of sex, age, and other risk factors [8, 9].

Metformin, an antidiabetic drug, has been shown to reduce insulin resistance (IR) and improve insulin sensitivity [10]. In a meta-analysis of randomized controlled trials (RCTs), a reduction in weight and calculated IR was observed in metformin users [10]. Metformin has several modes of action which involve AMP-activated protein kinase (AMPK)-dependent and AMPK-independent mechanisms that may be involved in LVH [11]. Metformin has also been shown to reduce LVH in animal models [12]. Observational studies have also reported beneficial cardiovascular effects for metformin in patients with type 2 diabetes mellitus (T2DM) and heart failure (HF) [13].

Two RCTs showed that metformin use improved LVMI in non-diabetic and pre-diabetic patients with pre-existing cardiovascular disease [14, 15]. Mohan and colleagues showed that the use of metformin for one year could regress LVMI in pre-diabetic patients or those with IR who have CAD and LVH [14]. Other RCTs showed that metformin use in heart failure with reduced ejection (HFrEF) patients improves myocardial oxygen efficiency [16, 17]. However, these two trials did not show a significant effect for metformin on LVEF, although the improvement in LVEF was greater in the intervention group than in the control group. In patients with metabolic syndrome, metformin use resulted in greater improvement in LVEF compared to using a placebo [18].

The scarcity of literature, and the low power to detect a statistically significant difference in LVMI and other left ventricular parameters were the key motivators for the current study. We hypothesized that metformin use could reduce LVMI and improve certain left ventricular parameters such as LVEF in non-diabetic patients. The current review critically evaluated the existing literature regarding metformin use in non-diabetic patients. A meta-analysis was also performed to test the research hypotheses.

## Methods

This meta-analysis was reported in accordance with the Preferred Reporting Items for Systematic reviews and Meta-Analyses (PRISMA) Statement. The protocol was registered with the PROSPERO registry (number CRD42021239368).

### Search strategy

The study was conducted in accordance with the PRISMA guidelines for systematic reviews. PubMed, Scopus database, Cochrane library, Clinicaltrials.gov, MEDLINE, medRxiv, and the WHO International Clinical Trials Registry Platform were searched for completed clinical trials published in any language evaluating the effect of metformin in non-diabetic patients with or without pre-existing cardiovascular disease. We used the search terms ("left ventricle" OR “LV dysfunction” OR “left ventricular dysfunction” OR LVMI OR "ejection fraction") AND (pre-diabetes OR non-diabetic OR "insulin resistance") AND randomized. The detailed search strategy for each of these databases can be found in Additional file 1: Table S1.

### Study outcomes

The primary outcome of the meta-analysis was the change in LVMI. Secondary outcomes were left ventricular ejection fraction (LVEF), NT-ProBNP or BNP, E/e′ ratio, global longitudinal strain (GLS), and left ventricular end-diastolic volume (LVEDV). E/e′ ratio was included as a measure of diastolic function. The analysis was performed using two approaches; First, the analysis was stratified by treatment duration to reduce heterogeneity. However, another analysis was performed using only the final time point in the study.

### Eligibility criteria

Studies were included if they met the following criteria: (1) RCT, (2) non-diabetic patients with pre-existing cardiovascular disease (STEMI, CAD, and HF) or without pre-existing cardiovascular disease (CVD) such as IR, pre-diabetes, or metabolic syndrome, (3) metformin only or metformin plus the standard of care (SOC) as the intervention (4) The control arm received only SOC or placebo, (5) Short term study duration (3–12 months). The following studies were excluded: (1) Studies that included diabetic patients, (2) observational (prospective or retrospective) clinical trials, and (3) Studies that did not assess any of the primary or secondary outcomes of interest.

### Quality assessment and risk of bias

For each trial, the risk of bias (“low risk,” “some concerns,” or “high risk” of bias) in the overall effect of metformin on ejection fraction and LVMI was assessed using version 2 of the Cochrane Risk of Bias Assessment Tool [19]. The tool is structured into five domains through which bias might be introduced into the result. We assessed the risk of bias in the estimated effect of adhering to the intervention (metformin therapy) as specified in the trial protocol (the ‘per-protocol effect’).

The risk of bias was assessed using published trial protocols and Consolidated Standards of Reporting Trials (CONSORT) flowcharts reported in the included studies. Other sources for risk of bias assessment included the methods used to generate and conceal the allocation sequence, blinding (single or double-blinded), the methods used to ensure that patients received their allocated intervention, the extent of deviations from the intended intervention, and the methods used to measure LVMI and LVEF. When applicable, authors of the included trials were contacted for needed missing information. The risk of bias assessment was done independently by two investigators (A.K and N.M), and disagreements were resolved through discussion and consulting with a third author (S. F).

### Data extraction

Two reviewers (A.K and N.M) screened and agreed on the included studies and assessed study bias, with a third reviewer as arbitrator (S.F). Full-text papers for the eligible studies were retrieved. Quantitative data were extracted from the included studies by one reviewer (A.K) and cross-checked by another reviewer (N.M) for completeness and accuracy. Extracted data included: study design, first author, country, publication year, subject characteristics (age and gender and comorbidities), intervention, dosage, dosage form, treatment duration, sample size, and the mean ± SD for the outcomes of interest.

### Effect size calculation

The standardized mean change using change score standardization (SMCC) was used to measure the effect in the current meta-analysis and was calculated as previously described [20]. The SMCC was used due to the variability in methods of assessing left ventricular function, heterogeneity of the study populations, the difference in standardization methods for LMVI (g/m^2^ vs. g/h^1.7^), the difference in methods for assessing left ventricular stretching (BNP vs. NT-ProBNP) which could bias the results if the unstandardized mean difference (MD) was used.

In brief, the standardized mean change was first calculated for each of the intervention and control groups (gT and gC, respectively) along with the sampling variance within each group. The effect size (g) for each study was then calculated as the difference between the two standardized mean change values (gT–gC). The corresponding sampling variances were calculated by adding up the sampling variances of the two groups:

Several methods were used to impute missing standard deviation (SD). The SD was calculated from the standard error (if reported) using the following formula: $= \frac{SD}{{\sqrt n }}$ (Additional file 1: Table S5c). For studies that reported median and interquartile range (IQR), the mean and standard deviation were estimated using the formulas suggested by Luo and Wan, respectively [21, 22], which are available using an online free calculator (https://www.math.hkbu.edu.hk/~tongt/papers/median2mean.html). In addition, the SD for the change in each group was estimated using the mean difference and 95% CI for the difference in change between groups or using the pre-test and post-test SD. A correlation of 0.7 was assumed between pre-treatment and post-treatment data. Previous research showed correlation values of 0.7–0.9 between pre-test and post-test echocardiographic parameters at 3–12 months in HF patients, with higher values observed at shorter follow-up time [16, 23, 24]. The correlation coefficient was either reported or estimated based on the formula written in Additional file 1: Table S4. Thus, the lower bound of 0.7 was used to avoid underestimating the variance and corresponding standard error.

The I^2^ statistic was used to explore the percentage of heterogeneity attributed to variation in true-effect sizes secondary to inter-population variation. Cochrane’s Q statistic was used to test the. Estimates from subgroups within the same study were pooled using a fixed-effects model and used in the meta-analysis. The 95% confidence interval (CI) and Z-statistic were calculated and used for hypothesis testing.

### Meta-analysis

Statistical analysis was performed using R v 3.6.3. The random-effects model was used to pool the effect sizes from different studies. The underlying hypothesis for adopting the random-effects model is that heterogeneity or observed variance of effect is a sum of sampling error and variation in true-effect sizes stemming from inter-population variability. The generic inverse variance method was used for weighting, and the Paule-Mandel (PM) was used as a heterogeneity variance estimator. The analysis was stratified by treatment duration (0–6 months, 7–12 months, and > 12 months) to reduce bias when combining studies with high variability in treatment duration. The analysis was also performed irrespective of treatment duration using the final time point from studies that reported the effect size at different time points. Forest plots were used to visualize the results. The effect size was estimated using the per-protocol population of each trial as some trials did not report the results using intention to treat (ITT) analysis. P values < 0.05 were considered statistically significant.

### Estimation of absolute difference

SMCC and SMCR were back-transformed to the original scale by multiplying the pooled estimates and the corresponding 95% CI by the SD of the change and the baseline scores, respectively [25].The resulting estimates allow a more intuitive interpretation of the results. The average pooled SD from the included trials was used for back-transformation.

### Sensitivity analysis

Sensitivity analysis was performed to assess the robustness of the results. The meta-analysis was repeated using the standardized change score using raw score standardization (SMCR) as Becker described [26], assuming a correlation of 0.7 based on previous literature (Additional file 1: Table S4). The baseline scores were used for standardization. The leave-one-out method was also to assess the robustness of the results and possible sources of heterogeneity.

The SMCR was tested for robustness by randomly sampling correlation coefficients between 0.6 and 0.9 for each arm of the included studies. The range of correlation values was decided after examining different correlation values reported in previous studies (Additional file 1: Tables S5a, b). The procedure was repeated 10,000 times. The lower or upper limit of the 95% CI (based on the direction of the effect) was calculated in each of these scenarios to ensure that the reported result does not change by varying the correlation coefficients.

### Publication bias

Funnel plots were used to assess publication bias. Egger’s test was used to test the asymmetry of funnel plots [27]. The trim-and-fill method was also used to detect and adjust for publication bias [28]. However, it has been suggested that the trim and fill method can underestimate the true positive effect when there is large between-study heterogeneity in the absence of publication bias due to variability in the true effect size [29]. We used the method suggested by Pustejovsky and Rodgers when testing for the funnel plot asymmetry as the effect is dependent on the standard error [30].

### Subgroup analysis and meta-regression

Meta-regression using robust variance estimation (RVE) was used to assess the effects of treatment duration, metformin dose (> 1000 mg), and presence of HF. RVE with small-sample correction was used to estimate correlated effects models using the original RVE methods to account for the presence of more than one time point for the same study [31]. RVE is distribution-free and provides valid point estimates, standard errors, and hypothesis tests even when the degree and structure of dependence between effect sizes are unknown. Meta-regression was also used to investigate these factors as possible sources of heterogeneity. An intercept-only model was fitted to assess the overall effect of metformin. Subgroup analysis was performed using intercept-free models to investigate the effect of metformin on the standardized change (SMCC) in each subgroup.

## Results

### Study selection

**Fig. 1:**
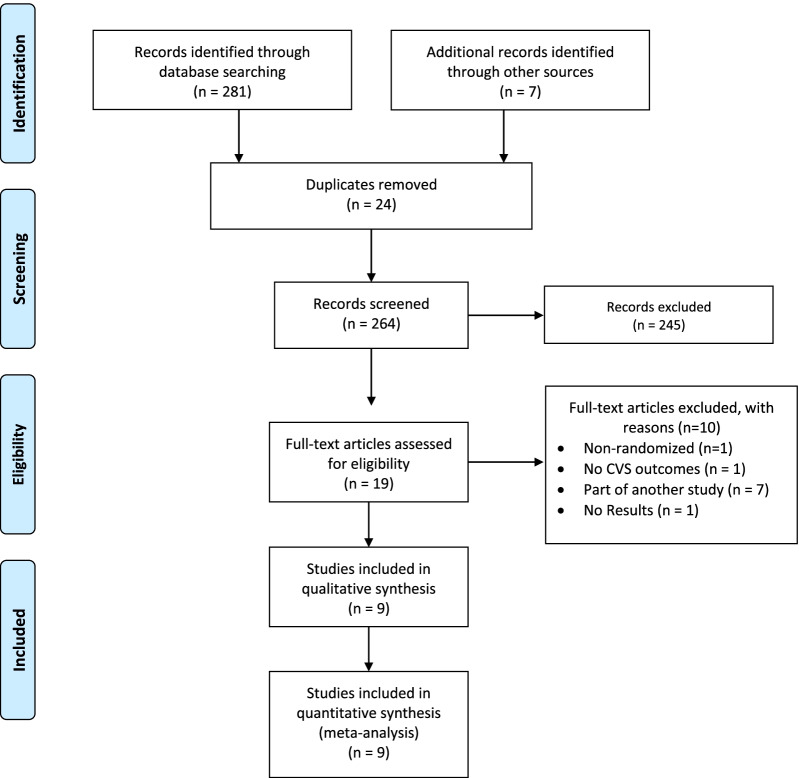
PRISMA diagram for study selection

The database search on the 5th of November returned 288 studies (Fig. 1). After screening, 24 duplicates were removed, and 264 studies were checked for eligibility. Of these, 245 were excluded. The full text for the remaining 19 articles was retrieved, and ten studies were excluded. Details regarding search results and excluded studies can be found in Additional file 1: Tables S2 and S3. Thus, nine RCTs were included in the current systematic review and meta-analysis [14–18, 32–35].

### Characteristics of the included studies

Four studies included patients with chronic stable HF; two included only HFrEF patients [16, 17], one included a combination of patients with HFrEF, heart failure with mid-range ejection fraction (HFmrEF), and heart failure with preserved ejection fraction (HFpEF) [33], and one included only patients with diastolic HF [15]. Two studies included patients with diastolic dysfunction [15, 18], and one included only STEMI patients at baseline [32]. LVMI was assessed using two-dimensional (2D) transthoracic echocardiography (TTE) in all but one study which used cardiac magnetic resonance imaging (CMR) [14]. Only one study presented the results for the intention to treat (ITT) population [16], and another presented the results for the modified intention to treat (mITT) [14]. All studies presented the per-protocol analysis results. Seven of the included studies were registered on clinicaltrials.gov [14–17, 32–34], one of which was retrospectively registered [33].

Characteristics of the included studies are shown in Table 1. A total of nine studies with 795 patients were included in the current systematic review. The number of patients per arm ranged from 17 to 39 in seven studies. All but two studies provided follow-up data at only one time point. Metformin was prescribed for < 6 months in three studies [16, 17, 32], 12 months in three studies [14, 33, 34], and 6 months in one study [18]. Two studies [15, 35] provided follow-up data at two (12 and 24 months) and three (6, 12, and 24 months) time points, respectively. The cumulative dose of metformin was < 1000 mg daily in two studies, 1000 mg in one study, and > 1000 in the remaining six studies (Table 1). The distribution of males and females was heterogeneous, with the percentage of males ranging from 28 to 90%. The average age across the included studies ranged from 40 to 65 years. Seven studies were conducted in Europe, one in India and one in Mexico. One study [32] reported NT-ProBNP for the whole population (n = 379) while LVEF, E/e′, and LVMI were reported for patients with a pre-defined subgroup (patients with diastolic dysfunction at baseline and follow up, n = 237). For the meta-analysis, NT-ProBNP for the whole population was used, and the subgroup provided the data for the remaining three parameters. Baseline final, and change values of the included studies are shown in Table 2.

**Table 1:** Characteristics of the included studies (n = 9)

Author	Year	Study Design	Follow up	Intervention (mg)	Control	Cumulative dose	Patient population	Outcomes	Sample size Randomized [E/C]	Male% [E/C]	Mean age [E/C]	Country	Outcome assessment
Ali [32]	2016	DB-RCT	4 months	500 × 2	Placebo	1000	STEMI patients	LVEF (4 months), LVM, LVMI, and diastolic function (E, e′, E/e, and LAVI)	118/119	80.5/74.8	57.9/58.2	Netherlands	2D Echocardiography (Vivid 7)
Gupta [33]	2020	OL-RCT	12 months	1000 × 2	SOC	2000	Documented CAD (angiographically documented or a previous history of myocardial infarction/angina), verified HF functional class III, FIRI ≥ 2.5 by receiving basic therapy for CAD and CHF (HFrEF, HFmrEF and HFpEF)	LVEF, Incidence of MI, hospitalization for HF decompensating, all-cause death, and conversion from Prediabetic to diabetes. secondary outcomes: QoL, (MLHFQ), neurohormonal, lipid profile, renal function, Insulin, aldosterone, and Nt-proBNP, 6 MWT	39/37	72.9/43.6	62/62	India	Transthoracic echocardiography (iE33 xMATRIX | Philips)
Ladeiras-Lopes [15]	2021	OL-RCT	3, 6, and 12 months	1000 × 2	SOC	2000	Non-diabetic adults aged 40–65 years with non-diabetic MetS and diastolic dysfunction	e′ velocity, LVEF, LVMI (g/m^2^), Pro-BNP, QoL (SF-36), CPX (Peak Vo2), FIRI, CRP	27/27	46/64	51.2/52.4	Portugal	2D Echocardiography
Larsen [16]	2020	DB-RCT	3 months	1000 × 2	SOC	2000	Insulin-resistant chronic HF patients without diabetes	LVMI (g/m^2^), LVEF, QoL (MLHFQ), HBA1c, HOMA-IR, Pro-BNP, E/e ratio, GLS, Myocardial efficiency (WMI, MEE), CPX (resting and max Vo2), 6 MWT	19/17	89/71	68/61	Denmark	2D Echocardiography (Vivid E9 and E95, GE Healthcare, Horten, Norway)
Mohan [14]	2019	DB-RCT	12 months	1000 × 2	Placebo	2000	CAD with IR and/or pre-diabetes	LVMI (g/m1.7), LVEF, Wt, TBA derivatives, Pro-BNP, A1C, HOMA-IR, systolic BP	31/32	84/75	64.5/64.5	UK	Cardiac MRI (CMR)
Sardu [34]	2021	DB-RCT	12 months	850 × 2	Placebo	1700	Obese patients with pre-diabetes. All 83 patients underwent abdominoplastic surgery and, after treatment, received a hypocaloric diet	inflammatory/oxidative stress, miRs’ expression, and cardiovascular function (LVMI and LVEF)	28/27	28.6/33.3	42.5/41.8	Italy	2D Echocardiography
Stakos [35]	2005	DB-RCT	12, and 24 months	500 mg × 1	Placebo	500	Non-diabetic patients with IR	Insulin sensitivity, glucose tolerance, lipid profile, LVMI (g/m^2^), aortic distensibility, aortic PWV	59/97	24/26	40.5/41	Greece	2D Echocardiography M-Mode Echocardiography
Velázquez [18]	2016	OL-RCT	Six months	850 × 1	SOC	850	35–60 years, diastolic dysfunction, abdominal obesity, and MS diagnosed according to the ATP III criteria. At least three of the five diagnostic criteria of MS	Echocardiographic parameters, Lipid profile, BP, and CRP	20/20	46/46	44.5/44	Mexico	2D Echocardiography (Phillips model IE33)
Wong [17]	2012	DB-RCT	4 months	1000 × 2	Placebo	2000	Non-diabetic IR HF patients (FIRI ≥ 2.7)	CPX (Peak Vo2 was the primary outcome), FIRI, VE/VCO2 slope, BNP, LVEF, 6-min walk test, MLHFQ	39/23	89.7/95.6	64/68	UK	2D Echocardiography

*E* experimental group, *C* control group, *BP* blood pressure, *CAD* coronary artery disease, *MS* metabolic syndrome, *CRP* C reactive protein, *CPX* cardiopulmonary exercise testing, *FIRI* fasting insulin resistance index, *HFrEF* heart failure with reduced ejection fraction, *HFmrEF* heart failure with mid-range ejection fraction, *HFpEF* heart failure with preserved ejection fraction, *IR* insulin resistance, *PWV* pulse wave velocity, *QoL* quality of life, *OL-RCT* open-label randomized clinical trial; DB-RCT: Double-blinded randomized clinical trial, *LAVI* left atrial ventricular index, *LVEF* left ventricular ejection fraction, *LVMI* left ventricular mass index, *SOC* standard of care, *TBA* thiobarbituric acid derivatives

**Table 2:** Baseline, final, and change values for the included studies

Author	LVMI (g/m^2^)		LVEF (%)		NT-ProBNP		E/e′ ratio	
	E	C	E	C	E	C	E	C
Ali [32]	B: 89 (75, 105)	B: 85 (73, 101)	B: 52 (45, 59)	B: 52 (46, 57)	B: 79.5 (42, 179)	B: 68 (37, 177)	B;7.8 (6.7, 9.6)	B:7.1 (5.9, 9.7)
	F4: 88.4 (75.7, 100.9)	F4: 85.7 (73.6, 97.0)	F4: 55 (48, 60)	F4: 58 (53, 63)			F4:7.6 (6.4, 9)	F4:7.5 (6.2, 8.7)
	△4: − 2.7 (− 16.0, 6.9)	△4: 0.6 (− 13.5, 9.1)	△4: 3.2 (− 2.9, 7.6)	△4: 3.7 (0.3, 10.6)			△4: − 0.1 (− 1.3, 1.0)	△4:0.2 (− 1.3, 1.9)
Gupta [33]			B: 46.0 (36.0–56.0)	B: 43.0 (33.0–50.0)	B: 996 (333–1798)	B: 903 (464–1378)		
			F12: 51.0 (42.0–61.0)	F12: 45.0 (35.0–52.0)	F12: 517 (246–1219)	F12: 701 (476–1157)		
			△: NR	△: NR	△: NR	△: NR		
Ladeiras-Lopes [15]	B: 88.7 ± 23.4	B: 84.6 ± 18.9	B: 58.4 ± 3.8	B: 60.1 ± 3.6	B: 52 (29–78)	B; 42 (14–52)	B: 9.3 ± 1.9	B: 8.6 ± 1.9
	MITT △6: 4.50 (14.81)	MITT △6: 2.57 (15.43)			MITT △6: 7.38 (34.02)	MITT △6: 6.43 ± 22.4	MITT △6: − 0.69 ± 1.28	MITT △6: 0.28 ± 1.10
	△12: − 4.12 (17.06)	△12: − 0.20 (16.33)			△12: 3.25 (19.49)	△12: 3.32 ± 12.38	△12: − 0.31 ± 1.60	△12: 0.12 ± 1.28
	△ 24: − 0.29 (15.30)	△ 24: 3.45 (14.75)			△24: 9.71 (31.83)	△ 24:1.35 ± 32.86	△ 24: − 0.57 ± 1.61	△ 24: 0.02 ± 1.54
Larsen [16]	B: 98 ± 25	B: 92 ± 25	B: 36 ± 9	B: 39 ± 6	B: 353 [222–896]	B: 364 [94–744]	B: 12 [11–14]	B: 11 [9–14]
	F3: 97 ± 22	F3: 94 ± 29	F3: 37 ± 10	F3: 38 ± 11	F3: 442 [194–1190]	F3:357 [103–562]	F3: 11 [9–14]	F3: 11 [9–15]
	△3: NR	△3: NR	△3: NR	△3: NR	△3: NR	△3: NR	△3: NR	△3: NR
Mohan [14]	B; 48.7 ± 6.5	B; 46.0 ± 9.3	△12 (MITT): − 3.58 ± 7.9	△12 (MITT): − 3.53 ± 6.6	B: 957.8 ± 1029	B: 796.5 ± 1247		
	△12 (MITT): − 2.71 ± 2.31	△12 (MITT): − 1.34 ± 2.66	△12 (PP): − 4.11 ± 8.4	△12 (PP): − 3.90 ± 6.81	△12 (MITT):309 ± 1390	△12 (MITT):99 ± 475		
	△12 (PP): − 3.12 ± 1.95	△12 (PP): − 1.29 ± 2.67			△12 (PP): 376 ± 1479	△12 (PP): 70 ± 458		
Sardu [34]	B: 94.11 ± 22.13	B: 93.54 ± 21.88	B: 52 ± 7	B:52 ± 7				
	F; 56.13 ± 16.18	F; 79.81 ± 16.83	F12: 57 ± 5	F12: 54 ± 8				
	△12: NA	△12: NA						
Stakos [35]	B: 123 ± 23.6	B: 127 ± 29.2						
	△12: − 8.1 ± 3.4	△12: − 2.9 ± 2.9						
	△ 24: − 16.8 ± 7.0	△ 24: − 0.2 ± 4.1						
Velázquez [18]	B: 99.7 ± 20	B: 96 ± 26	B: 71 ± 55	B: 71 ± 40			B: 9.5 ± 2.5	B: 10.6 ± 2.3
	F6: 89 ± 18	F6: 98 ± 23	F6: 73 ± 58	F6: 68 ± 29			F6: 9.59 ± 2.1	F6: 10.4 ± 1.5
	△6: NR	△6: NR	△6: NR	△6: NR			△6: NR	△6: NR
Wong [17]			B: 34 ± 8	B: 30 ± 8	BNP	BNP		
			△4: 0.35 + 5.50	△4: − 1.10 + 4.20	B:131.7 ± 158.5	B: 187.1 ± 251.3		
					△4: − 20.2 ± 78.7	△4: 7.5 ± 131.2		

Data was presented as mean ± standard deviation or median [interquartile range]

B: Baseline, F3: Final value at 3 months, F4: Final value at 4 months, F6: Final value at 6 months, F12: Final value at 12 months

△3: Change at 3 months, △4: Change at 4 months, △6: Change at 6 months; △12: Change at 12 months, △24: Change at 24 months

C: Control group; E: Experimental group

*MITT* modified intention to treat, *NR* not reported, *PP* per protocol

^¶^LVMI was indexed to height^1.7^

### Risk of bias assessment

**Fig. 2:**
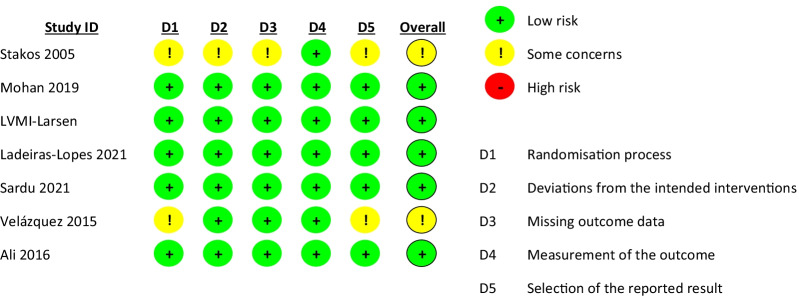
Risk of bias assessment in the effect of metformin on LVMI

The risk of bias in the estimated effect of metformin on LVMI (Fig. 2) was assessed as low in seven studies and having some concerns in the studies conducted by Stakos and Velázquez [18, 35]. The risk of bias was assessed as having some concerns in four and two domains, respectively. Both studies did not report the randomization process and did not have a pre-specified analysis plan or registered protocol. In addition, baseline line values reported by Stakos suggested bias in allocating patients to the treatment groups. Regarding LVEF, the risk of bias was assessed as low in seven studies and having some concerns in one. The risk of bias was high in one study due to probable lack of concealment of allocation, issues with baseline characteristics, and lack of a pre-defined analysis plan. Details regarding RoB assessment for LVMI and LVEF can be found in Appendices S2 and S3, respectively.

### Primary outcome

Seven studies with 625 patients provided data regarding the change in LVMI (292 and 333 in the metformin and control arms, respectively). The LVMI was standardized as g/h^1.7^ in one study [14], and all the remaining studies provided LVMI indexed to body surface area (g/m^2^).

**Fig. 3:**
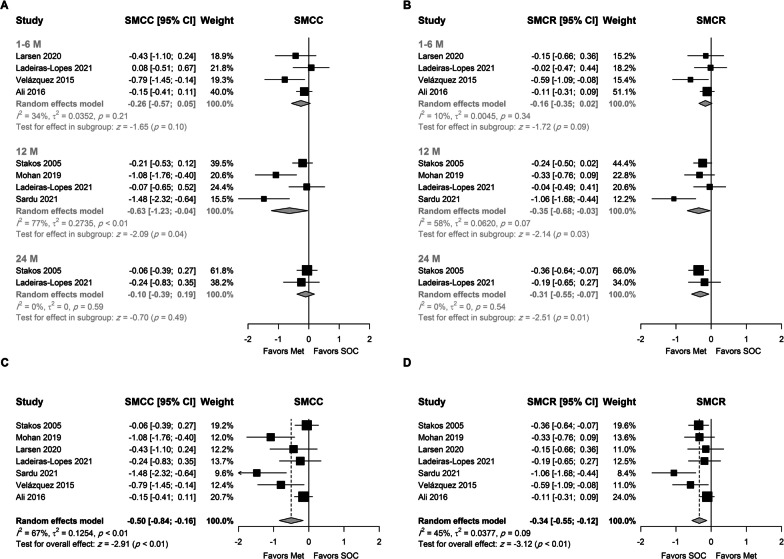
Random-effects model for the association between metformin and LVMI using **A** SMCC stratified by treatment duration, **B** SMCR stratified by treatment duration, **C** SMCC using final time point, and **D** SMCR using the final time point

Results (Fig. 3A, B) showed that metformin had a favorable effect on LVMI after 12 months using SMCC (SMCC = −0.63, 95% CI − 1.23; − 0.04, p = 0.04) and SMCR (SMCR = −0.35, 95% CI − 0.65; − 0.04, p = 0.03) as measures of effect size. Assuming a pooled SD of 18 and 22 g/m^2^ for SMCC and SMCR, the above values can be interpreted as an absolute reduction of 11.3 (95% CI 22.1–0.72) g/m^2^ and 7.59 (14.1–0.87) g/m^2^, respectively.

A favorable effect of metformin was also present at six months, although it did not reach statistical significance (p = 0.1 and 0.09 for SMCC and SMCR, respectively). At two years, a meta-analysis of two studies showed that the change in LVMI was greater in patients who received metformin when the SMCR was used as the unit of analysis (SMCR = −0.31, 95% CI − 0.55; − 0.07, p = 0.01). These values correspond to a higher absolute reduction of 6.72 (95% CI 11.93–1.52) g/m^2^ in the metformin arm. The trim-fill method and Egger's test were not used due to the small number of studies in each subgroup.

Analysis using the last time point in each study (Fig. 3C, D) showed a statistically significant favorable effect for metformin irrespective of the measure of effect. The reduction in LVMI was higher in the metformin group by 0.5 SD (SMCC = −0.5, 95% CI − 0.84; − 0.16, p < 0.01) and 0.33 SD (SMCR = −0.33, 95% CI − 0.53; − 0.13, p < 0.01) than the control group. The SMCR was robust to change in the correlation coefficient. These values correspond to a higher absolute reduction of 8.97 (95% CI 15.06–2.87) and 7.18 (95% CI 11.49–2.82) g/m^2^ in the metformin arm, respectively.

Substantial heterogeneity in the observed effect size was observed between studies (I^2^ = 67% and 45% for SMCC and SMCR, respectively). The studies conducted by Sardu [34] and Ali [32] were identified as potential outliers. However, the results were robust to leave-one-out sensitivity analysis (Suppl. Figures 1 and 2). No heterogeneity was observed using SMCR as the unit of analysis (Suppl. Figure 2) when the study conducted by Sardu was omitted (SMCR = −0.23, 95% CI − 0.36: − 0.1, I^2^ = 0%). In either case, funnel plots were symmetric around the calculated effect size (Suppl. Figure 3). Egger’s test was not statistically significant in either case (p = 0.12 and 0.24, for SMCC and SMCR, respectively).

### Secondary outcomes

#### Left ventricular ejection fraction

**Fig. 4:**
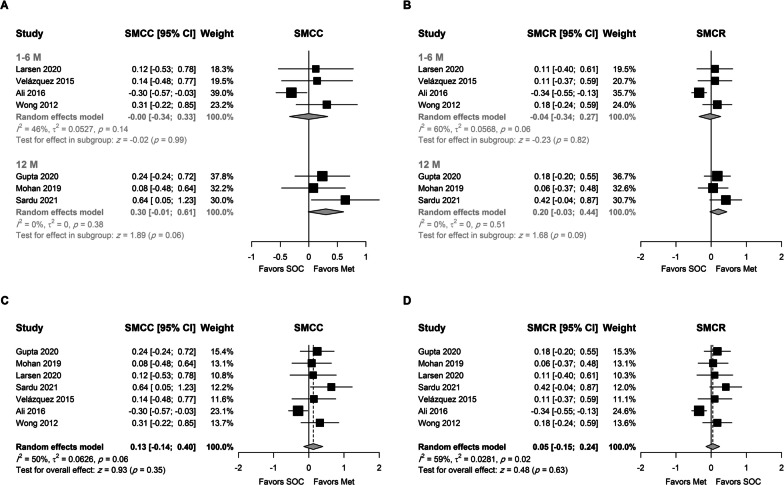
Random-effects model for the association between metformin and LVEF using **A** SMCC stratified by treatment duration, **B** SMCR stratified by treatment duration, **C** SMCC using final time point, and **D** SMCR using the final time point

Seven studies, which included 553 patients, provided change values for LVEF. Baseline SD was not reported in one study, and the SD was imputed by multiplying the change SD by 1.5, which was estimated from baseline and change SD values in the included studies. No association was observed between the use of metformin and the improvement in LVEF (as SMCC or SMCR), although the average increase in LVEF was higher by 0.3 and 0.2 SD at 12 months, respectively (Fig. 4A, B). However, it did not reach the statistical significance at the 0.05 level (P = 0.06 and 0.09, respectively). None of the included studies assessed the effect of metformin on LVEF at 24 months.

Using the final time point, the pooled analysis did not reveal a beneficial effect for metformin on LVEF (Fig. 4C, D). Moderate to substantial heterogeneity was observed, and further analysis showed that such heterogeneity was attributed to the study conducted by Ali. Interestingly, its removal (Suppl. Figures 4 and 5) resulted in a statistically significant effect for metformin on LVEF and no heterogeneity (I^2^ = 0) between studies (SMCC = 0.26, 95% CI 0.03–0.49, P = 0.03, and SMCR = 0.17, 95% CI 0.00–0.35, P = 0.05). The use of the trim-fill method did not affect the results (Suppl. Figure 6). These values were back-transformed to a higher absolute average increase of 2.99% (95% CI 0.34; 5.63) and 2.54% (95% CI 0; 5.08), in the metformin arm, respectively.

#### E/e*′* ratio

**Fig. 5:**
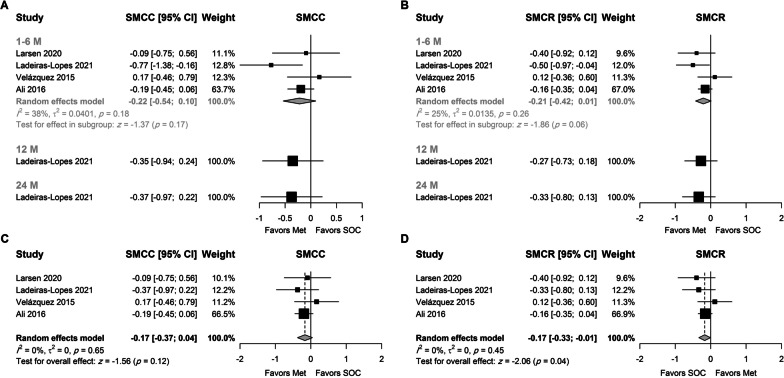
Random-effects model for the association between metformin and E/e′ ratio using **A** SMCC stratified by treatment duration, **B** SMCR stratified by treatment duration, **C** SMCC using final time point, and **D** SMCR using the final time point

Only four studies provided data regarding E/e′ ratio change, two of which included only patients with diastolic dysfunction (Fig. 5). When the analysis was stratified by treatment duration (Fig. 5A, B), metformin was not associated with the change in E′/ratio regardless of the measure of effect size. Low and non-statistically significant heterogeneity was observed at six months (I^2^ = 38% and 25% for SMCC and SMCR, respectively).

Nonetheless, the use of metformin was associated with an overall higher reduction in E/e′ ratio (SMCR = −0.17, 95% CI − 0.33 to − 0.01, P = 0.04). The results were not robust to leave-one-out sensitivity analysis, and only the SMCR was statistically significant when only one of the four included studies were omitted (Suppl. Figures 7 and 8). Only the funnel plot for the SMCC was asymmetric around the pooled estimate (Suppl. Figure 9a). Thus, the trim-fill method did not affect the pooled SMCR estimate. However, the pooled SMCC was statistically significant after using the trim-fill method (Suppl. Figure 9b), supporting the results obtained using SMCR (SMCC = −0.21, 95% CI − 0.4 to − 0.01, P = 0.04). Furthermore, no heterogeneity was observed between studies (I^2^ = 0%).

#### BNP or NT-ProBNP

**Fig. 6:**
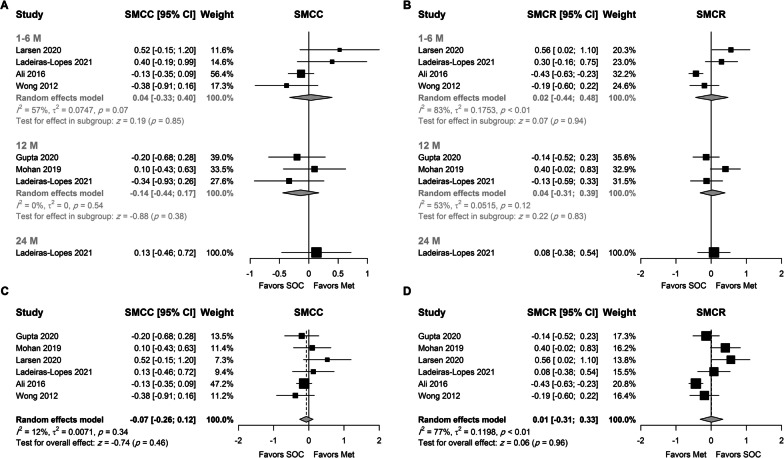
Random-effects model for the association between metformin and NT-ProBNP/BNP using **A** SMCC stratified by treatment duration, **B** SMCR stratified by treatment duration, **C** SMCC using final time point, and **D** SMCR using the final time point

Five studies provided data regarding the change in NT-ProBNP, and one study provided only BNP data. Metformin was not associated with the change in NT-ProBNP/BNP irrespective of the treatment duration or the analysis methods (Fig. 6). The analysis was robust to leave-one-out sensitivity analysis (Suppl. Figures 10 and 11), and no to minimal heterogeneity was observed for the pooled SMCC at 12 months (Fig. 6A) and the overall pooled SMCC (Fig. 6C). None of the individual studies was identified as a sole source of heterogeneity for the overall pooled SMCR (Suppl. Figure 11).

#### Meta-regression analysis

Meta-analysis using RVE (Table 3) was performed to assess the overall effect of metformin on the standardized effect size (intercept only model) as well as the effect of metformin in different subgroups (intercept-free models).

**Table 3:** Meta-regression analysis for the effect of metformin on SMCC of cardiovascular parameters and NT-ProBNP

	LVMI		LVEF		E/e′ ratio		NT-ProBNP/BNP	
	B [95% CI]	p	B [95% CI]	p	B [95% CI]	p	B [95% CI]	p
Intercept only model	− 0.49 [− 0.94; − 0.04]	**0.036**	0.128 [–0.19; 0.45]	0.45	–0.18 [− 0.387; 0.03]	**0.07**	− 0.07 [− 0.28; 0.14]	0.44
Metformin dose								
1000 mg or less	− 0.287 [–0.7; 0.134]	0.14	–0.23 [− 0.49; 0.03]	0.07	− 0.128 [− 0.59; 0.34]	0.36	NA	
> 1000 mg	− 0.7 [− 1.51;0.11]	0.08	0.28 [0.04; 0.52]	**0.04**	− 0.313 [− 1.2; 0.56]	0.26	− 0.02 [− 0.44; 0.41]	0.92
Duration								
6 months or less	− 0.215 [− 0.51; 0.08]	0.12	− 0.02 [− 0.48; 0.43]	0.9	− 0.163 [− 0.5; 0.17]	0.17	− 0.05 [− 0.5; 0.41]	0.79
> 6 months	− 0.4 [− 0.78; − 0.02]	**0.04**	0.3 [− 0.07; 0.68]	0.09	NA		− 0.07 [− 0.36; 0.21]	0.53
HF								
No	− 0.51 [− 1.1; 0.08]	0.09	0.06 [− 0.5; 0.62]	0.81	− 0.19 [− 0.73; 0.36]	0.28	− 0.04 [− 0.26; 0.19]	0.68
Yes	NA		0.23 [0.1; 0.36]	**0.004**	NA		− 0.08 [− 0.73; 0.56]	0.74
Pooled baseline average (1 unit increase)	0.005 [− 0.02; 0.03]	0.64	− 0.005 [− 0.02; 0.01]	0.34	0.05 [− 0.13; 0.23]	0.35	0.09 ¶ [− 0.05; 0.06]	0.69

Bold values denote statistical significance at the *p* < 0.05 level

B—regression coefficient (SMCC), CI.L—lower limit for 95% confidence interval, CI.U—upper limit for the 95% confidence interval

*HF* heart failure

NA: Only one study provided data

^¶^Result represents the change in SMCC for each 100-unit increase

Meta-regression (Table 3) analysis revealed a statistically significant overall favorable effect for metformin on LVMI (SMCC = −0.49, 95% CI 0.1: − 0.94; − 0.04, P = 0.04) which translates to a higher absolute average reduction of ~ 9 g/m^2^. The effect of metformin on LVMI was statistically significant in patients who received metformin for > 6 months (SMCC = −0.4, 95% CI − 0.78; − 0.02, P = 0.04). The overall reduction in E/e′ ratio was also higher in patients who received metformin although it did not reach statistical significance at the 0.05 level (SMCC = −0.18; 95% CI − 0.39; 0.03, P = 0.07).

**Fig. 7:**
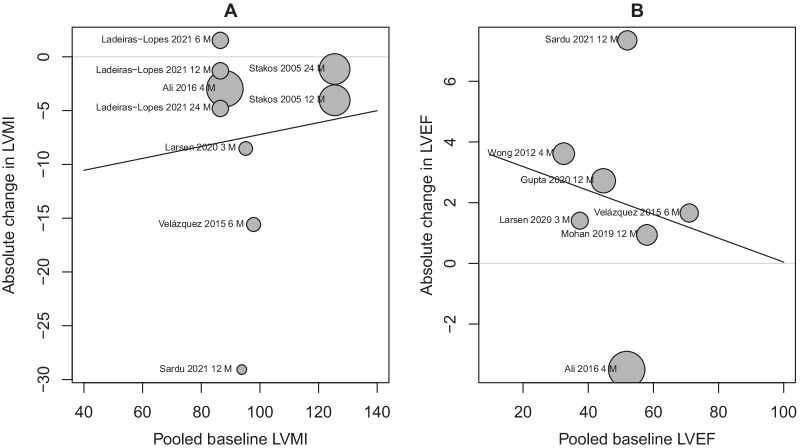
Bubble plot for the association between **A** baseline LVMI and absolute change (g/m^2^) in LVMI **B** baseline LVEF and absolute change (%) in LVEF. Absolute values were obtained by multiplying the SMCC for LVMI and LVEF by the pooled average SD of 18 mg/m^2^ and 11.5%, respectively

A favorable effect for metformin was observed on LVEF only in patients who received > 1000 mg/day (SMCC = 0.28, 95% CI 0.04; 0.52, P = 0.04), and patients with HF (SMCC = 0.23; 95% CI 0.1; 0.36, P = 0.004). These values correspond to absolute differences of 2.64% and 3.21%, respectively. No association was observed between baseline values and the SMCC for any of the included parameters. Bubble plots (Fig. 7) show the association between baseline values of LVMI and LVEF and the difference in the absolute change (g/m^2^ and %, respectively).

## Discussion

Metformin is a commonly prescribed drug for the treatment of diabetes, used by millions of patients worldwide daily, including patients with HF [36, 37]. The current meta-analysis investigated the role of metformin in non-diabetic patients and showed a favorable effect for metformin on LVMI after 12 months of use (higher reduction of ~ 10 g/m^2^). Metformin use was associated with a modest but higher improvement in LVEF (2–3%). Further subgroup analysis revealed a favorable effect for metformin on LVEF in patients who received > 1000 (absolute increase of 2.64%) and HF patients (absolute increase of 3.21%).

The favorable and long-lasting benefits of metformin on CV morbidity and mortality, in addition to its good safety profile, have further supported its use. A recent meta-analysis of 33 studies including 61,704 patients showed that metformin use was associated with a lower incidence of coronary revascularization, cardiovascular and all-cause mortality, and HF in patients with pre-existing CVD [38]. However, it was not associated with a reduction in the incidence of myocardial infarction, angina, and stroke. Another meta-analysis showed that metformin reduced all-cause and cardiovascular mortality in diabetic and non-diabetic patients with coronary heart disease [39]. However, most of the published literature included observational studies, which are liable to confounding and none of them included enough RCTs to appropriately investigate the effect of metformin primarily on LVMI or LVEF.

In terms of clinical significance, the pooled estimate for LVMI translated to an absolute reduction of ~ 10 g/m^2^. Evidence has shown that the regression of LVH was associated with a lower incidence of major cardiovascular events irrespective of BP changes [40, 41]. The results from the LIFE study showed one SD reduction in LVMI was associated with a 26% (95% CI 7–41%) reduction in all-cause mortality after adjusting for different covariates. Extrapolating these results to the current study indicate that a reduction of 0.35 SMCR is predicted to produce a ~ 9.1% reduction in all-cause mortality [42].

Of note, all of the included studies showed a positive effect for metformin on LVM, with the effect being statistically significant in three of them [14, 18, 34]. The sample size in most of the included studies was small (~ 25 per group), which might have increased the chance of type II error. Moreover, all but one study used TTE to assess LVMI. The use of CMR to assess LVMI could partly explain the statistically significant effect in Mohan’s study, as it is more sensitive to change than TTE [43]. Furthermore, the study included only patients with LVH at baseline and standardized the LVMI to height rather than BMI. The change in LVMI can be regarded as a surrogate marker for CV mortality, as suggested by several studies that showed a higher risk of morbidity and mortality in patients with LVH [44–46]. Thus, regression of LVH by using metformin might positively affect morbidity and mortality, ultimately improving the quality of life of these patients.

The results of the current meta-analysis also showed a beneficial effect for metformin on LVEF only when the study conducted by Ali (GIPS-III trial) was removed from the analysis [47]. This might be explained by the study population (STEMI patients), the short duration of therapy (4 months), and the low metformin dose (1000 mg/day). Moreover, the effect of metformin might have been influenced by the disease prognosis rather than metformin.

The modest improvement in LVEF observed in the current meta-analysis may not be relevant in the clinical setting especially in patients with normal baseline LVEF. This improvement is lower than the suggested clinical threshold reported in the STICH trial which included ischemic cardiomyopathy patients. The results showed that a 10% improvement in LVEF, although not common, is associated with a 40% reduction in the hazard of all-cause mortality at two years [48].

The results should be interpreted with caution due to the variability in baseline values between studies, duration, and dose regimens of metformin. This does not allow a robust conclusion regarding the exact duration or dose beyond which a beneficial effect is expected. Nonetheless, exploratory meta-regression indicated that HF patients are more likely to benefit from such therapy. In addition, the SMCC was statistically significant when higher doses and longer duration of therapy were used.

Research has shown that metformin can reduce infarct size and improve LVEF in HF models post-MI [49, 50], and further research showed that these effects were mediated via AMPK-eNOS-mediated signaling [51].

A systematic review of nine RCTs suggested that metformin can improve HF-related outcomes in IR or T2DM patients, especially in individuals without overt signs of CVD [52]. These results are in line with the conclusions of the current meta-analysis, which showed that metformin has a favorable effect in HF patients. In the EMPA-REG trial, diabetic patients with established CV disease who were treatment naïve or on stable glucose-lowering therapy (98% of the patients) were randomized to either placebo or empagliflozin. At baseline, 74% of patients were using metformin. Post-hoc subgroup analysis (based on metformin use) showed a higher risk of CV death, mortality, and nephropathy in the placebo arm of the metformin non-users than metformin users suggesting that metformin use might attenuate the effect of sodium-glucose transporter (SGLT-2) inhibitors on these outcomes [53]. The lower effect of empagliflozin might even be attributed an underlying protective effect of metformin which attenuated the effect of empagliflozin although such hypothesis requires further evaluation before conclusions can be drawn as a possible result of selection bias driven by the kidney function.

Historically, metformin was contraindicated in patients with HF due to concerns over the development of life-threatening lactic acidosis and was ultimately withdrawn from the US market [54]. However, evidence from a large observational study showed that HF patients who were on metformin and followed up for an average of ten years did not observe hospitalizations or deaths due to lactic acidosis [55] and a review of existing evidence further supported these results [56]. Two of the studies in the current analysis included only patients with HFrEF, and lactic acidosis was not reported in either [15, 16]. In a systematic review of observational studies, metformin use was not associated with higher risk of lactic acidosis in HF patients compared to other antidiabetic medications and was associated with lower risk of mortality [57]. Another systematic review suggested that the cautions use of metformin, with appropriate follow up and dose adjustment, could be expanded to patients with mild to moderate kidney impairment [58].

Regarding E/e′ ratio, a trend towards a positive effect was observed in the current meta-analysis. However, the results were not robust to various sensitivity analyses. The low number of studies (n = 4) included in the E/e′ ratio meta-analysis can explain the lack of a decisive conclusion. Further studies are needed to augment the current meta-analysis results to conclude whether metformin can be added to the standard regimen of patients with HFpEF. Another systematic review explored the effect of metformin in T2DM patients with HFpEF and showed that it could lower mortality in the long run [59].

The mechanisms which explain the above findings are complex and involve more than one pathway. Direct and indirect mechanisms can explain the protective role of metformin. Metformin has been shown to directly affect the myocardium by increasing myocardial energy metabolism and efficiency by activating AMPK which improves glucose utilization, mitochondrial respiration, and ATP synthesis in cardiomyocytes, ultimately leading to better systolic and diastolic effectiveness [50, 60, 61]. Metformin counteracts oxidative stress, which affects mitochondrial function, increasing NO synthesis, and other pathways [62]. Irrespective of glycemic status, metformin has direct potent anti-remodeling properties through reducing myocardial hypertrophy and fibrosis, thus, preserving LV morphology [63]. Indirectly, Metformin positively affects the development and progression of atherosclerosis in type I diabetic patients [64] and has been shown to modestly reduce blood pressure in non-diabetic patients, which can lower the risk of cardiovascular events [65].

Nonetheless, All the included studies failed to show a beneficial effect of Metformin on BNP or NT-ProBNP, and the heterogeneity of baseline values might have confounded any beneficial effect as most studies included patients with normal BNP or ProBNP at baseline. Regarding other outcomes, only one and two studies provided data regarding GLS and LVEDV, respectively. Thus, a meta-analysis of these outcomes was not possible. Several randomized studies such as the MET-HEFT (NCT03514108), VA IMPACT (NCT02915198), and GLINT (ISRCTN34875079) trials are currently ongoing to study the CV outcomes of metformin use, and their results should provide more evidence to support the use of metformin in patients at high risk of negative CV events.

### Limitations

The current study had several limitations. First, only nine randomized clinical trials were included, and stratifying the analysis by various factors might not have yielded enough power to detect a statistically significant difference in different subgroups when the analysis was stratified by treatment duration or when meta-regression was performed. For example, meta-analysis for the standardized change in E/e′ ratio included only four studies. Nonetheless, there was clear evidence regarding the beneficial effect of metformin on LVMI and LVEF in patients with HF. Thus, subgroup analysis and meta-regression could be regarded as exploratory. There was also some degree of heterogeneity in the included studies due to the variability in the patient population across studies, treatment duration, and dose regimen. We also did not investigate the long-term effects of metformin on all-cause mortality and morbidity as they were not the primary focus of the study.

## Conclusion

Results from the current review suggest a favorable effect for metformin on LVMI in patients with or without pre-existing CVD. A longer duration of metformin was associated with a higher effect. Metformin use was associated with a modest improvement in LVEF, and these results were further demonstrated in a subgroup of HF patients. Additional trials are needed to address the long-term effect of metformin.

## Supplementary Information

**Additional file 1.** Search Strategy, Calculation Formulas, and Sensitivity Analysis.

**Additional file 2.** Risk of Bias Assessment in the Effect ofMetformin on Left Ventricular Mass Index (LVMI).

**Additional file 3.** Risk of Bias Assessment in the Effect ofMetformin on Left Ventricular Ejection Fraction (LVEF).

## Data Availability

The datasets used and/or analyzed during the current study are available from the corresponding author on reasonable request.
