# Supplementary material for: Effect of metformin on left ventricular mass and functional parameters in non-diabetic patients: a meta-analysis of randomized clinical trials
Source: BMC Cardiovasc Disord. 2022 Sep 10;22:405. doi: 10.1186/s12872-022-02845-w (PMC9464374; doi:10.1186/s12872-022-02845-w)
Supplement: Supplementary file 1 — Additional file 1. Search Strategy, Calculation Formulas, and Sensitivity Analysis. [file 12872_2022_2845_MOESM1_ESM.pdf]

Effect of Metformin on Left Ventricular Mass and Functional Parameters in Non-Diabetic Patients: A Meta -analysis of Randomized Clinical Trials

Ahmed M. Kamel, MSc [a], Nirmeen Sabry, PhD [a], Samar Farid, PhD [a]

[a] *Clinical Pharmacy Department, Faculty of Pharmacy Cairo University, Cairo, 11562, Egypt*

***Corresponding author**

Ahmed Mohamed Kamel, MSc

Cairo University, College of Pharmacy, Department of Clinical Pharmacy, Egypt.

E-mail addresses: ahmedm.kamel@pharma.cu.edu.eg

Tel: +010-0676-6275

Fax: +011-202-25320005

ORCID ID:

Ahmed M. Kamel: 0000-0002-3791-5998

Nirmeen Sabry: 0000-0003-0478-0772

Samar Farid: 0000-0002-6048-847X

Supplementary Material

Contents

1. Search Strategy 3
2. Reasons for Excluding Abstracts 6
3. Reasons for Excluding Full-Text Research 7
4. Calculation Formulas 9
5. Estimated Correlation Coefficients and Standard Deviations 10
6. Leave One-Out Sensitivity Analysis For LVMI 11
7. Funnel Plot Asymmetry for The Effect Of Metformin On LVMI 12
8. Leave One-Out Sensitivity Analysis for The Effect Of Metformin On LVEF 13
9. Funnel Plot Asymmetry for The Effect Of Metformin On LVEF 14
10. Sensitivity Analysis for The Effect Of Metformin On E/E' Ratio 15
11. Funnel Plot Asymmetry for The Effect Of Metformin On E/E' Ratio 16
12. Sensitivity analysis for the effect of metformin on NT-ProBNP/BNP 17
13. Funnel plot asymmetry for the effect of metformin on NT-ProBNP/BNP 18

List of Abbreviations:

LLCI	Lower limit of the 95% confidence interval
LVEF	Left ventricular ejection fraction
LVMI	Left ventricular mass index
SD	Standard deviation
SE	Standard error
SMCC	Standardized mean difference in change using change score
SMCR	Standardized mean difference in change using raw score
ULCI	Upper limit of the 95% confidence interval

1. Search Strategy

Table S1: Detailed search strategy for the included databases

**PubMed (Text Word) (5th of November)**		
#1	((((left ventricle) OR (LV dysfunction)) OR (left ventricular dysfunction)) OR (LVMI)) OR (ejection fraction)<br>heart ventricles"[MeSH Terms] OR ("heart"[All Fields] AND "ventricles"[All Fields]) OR "heart ventricles"[All Fields] OR ("left"[All Fields] AND "ventricle"[All Fields]) OR "left ventricle"[All Fields] OR ("LV"[All Fields] AND ("dysfunctional"[All Fields] OR "dysfunctionals"[All Fields] OR "dysfunctioning"[All Fields] OR "dysfunctions"[All Fields] OR "physiopathology"[MeSH Subheading] OR "physiopathology"[All Fields] OR "dysfunction"[All Fields])) OR ("ventricular dysfunction, left"[MeSH Terms] OR ("ventricular"[All Fields] AND "dysfunction"[All Fields] AND "left"[All Fields]) OR "left ventricular dysfunction"[All Fields] OR ("left"[All Fields] AND "ventricular"[All Fields] AND "dysfunction"[All Fields])) OR "LVMI"[All Fields] OR (("eject"[All Fields] OR "ejected"[All Fields] OR "ejecting"[All Fields] OR "ejection"[All Fields] OR "ejectional"[All Fields] OR "ejections"[All Fields] OR "ejects"[All Fields]) AND ("dose fractionation, radiation"[MeSH Terms] OR ("dose"[All Fields] AND "fractionation"[All Fields] AND "radiation"[All Fields]) OR "radiation dose fractionation"[All Fields] OR "fractionation"[All Fields] OR "chemical fractionation"[MeSH Terms] OR ("chemical"[All Fields] AND "fractionation"[All Fields]) OR "chemical fractionation"[All Fields] OR "fraction"[All Fields] OR "fraction s"[All Fields] OR "fractionate"[All Fields] OR "fractionated"[All Fields] OR "fractionates"[All Fields] OR "fractionating"[All Fields] OR "fractionationed"[All Fields] OR "fractionations"[All Fields] OR "fractionator"[All Fields] OR "fractionators"[All Fields] OR "fractioned"[All Fields] OR "fractioning"[All Fields] OR "fractionized"[All Fields] OR "fractions"[All Fields]))	234,608
#2	**((pre-diabetes) OR (non-diabetic)) OR (insulin resistance) OR (prediabetes)**<br>**prediabetic state"[MeSH Terms] OR ("prediabetic"[All Fields] AND "state"[All Fields]) OR "prediabetic state"[All Fields] OR ("pre"[All Fields] AND "diabetes"[All Fields]) OR "pre diabetes"[All Fields] OR "non-diabetic"[All Fields] OR ("insulin resistance"[MeSH Terms] OR ("insulin"[All Fields] AND "resistance"[All Fields]) OR "insulin resistance"[All Fields])**	186747
#3	**(randomized clinical trial) OR (randomized controlled trial) OR (rct) OR (randomized)**<br>**"random allocation"[MeSH Terms] OR ("random"[All Fields] AND "allocation"[All Fields]) OR "random allocation"[All Fields] OR "random"[All Fields] OR "randomization"[All Fields] OR "randomized"[All Fields] OR "randomisation"[All Fields] OR "randomisations"[All Fields] OR "randomise"[All Fields] OR "randomised"[All Fields] OR "randomising"[All Fields] OR "randomizations"[All Fields] OR "randomize"[All Fields] OR "randomizes"[All Fields] OR "randomizing"[All Fields] OR "randomness"[All Fields] OR "randoms"[All Fields]**	1,326,592
#4	(Metformin) OR (biguanide) OR (Glucophage)<br>metformin"[MeSH Terms] OR "metformin"[All Fields] OR "metformine"[All Fields] OR "metformin s"[All Fields] OR "metformins"[All Fields] OR "biguanid"[All Fields] OR "biguanides"[MeSH Terms] OR "biguanides"[All Fields] OR "biguanide"[All Fields] OR "biguanids"[All Fields] OR "metformin"[MeSH Terms] OR "metformin"[All Fields] OR "glucophage"[All Fields] OR "metformine"[All Fields] OR "metformin s"[All Fields] OR "metformins"[All Fields]	40510
#5	**((#1) AND (#2)) AND (#3) AND (#4)**	17
**Scopus (4th of November)**		
#1	left AND ventricle OR lv AND dysfunction OR left AND ventricular AND dysfunction OR lvmi OR ejection AND fraction	111608
#2	(pre-diabetes OR non-diabetic OR insulin AND resistance OR prediabetes)	657782
#3	(randomized AND clinical AND trial OR randomized AND controlled AND trial OR rct)	2850118
#4	(metformin OR biguanide OR Glucophage)	175245

#5	(left AND ventricle OR lv AND dysfunction OR left AND ventricular AND dysfunction OR lvmi OR ejection AND fraction) AND (pre-diabetes OR non-diabetic OR insulin AND resistance OR prediabetes) AND (randomized AND clinical AND trial OR randomized AND controlled AND trial OR rct) AND (metformin OR biguanide OR glucophage) AND (LIMIT-TO (DOCTYPE , "ar")) AND (LIMIT-TO (EXACTKEYWORD , "Human")) AND (LIMIT-TO (EXACTKEYWORD , "Humans"))	129
**Cochrane (All Text)(5th of November)**		
#1	(left ventricle) OR (LV dysfunction) OR (left ventricular dysfunction) OR (LVMI) OR (Ejection fraction) in All Text - in Trials (Word variations have been searched)	22819
#2	(pre-diabetes) OR (non-diabetic) OR (insulin resistance) OR (prediabetes)	20821
#3	(randomized clinical trial) OR (randomized controlled trial) OR (rct)	1276485
#4	(metformin) OR (biguanide) OR (glucophage)	11939
#5	(left ventricle) OR (LV dysfunction) OR (left ventricular dysfunction) OR (LVMI) OR (Ejection fraction) in All Text AND (pre-diabetes) OR (non-diabetic) OR (insulin resistance) OR (prediabetes) in All Text AND (randomized clinical trial) OR (randomized controlled trial) OR (rct) in All Text AND (metformin) OR (biguanide) OR (glucophage) in All Text - in Trials	**36**
**Clinicaltrials.gov (5th of November)**		
Final	Left ventricular dysfunction AND prediabetes AND Metformin AND interventional studies	**89**
**Medline (5th of November)**		
#1	(((TS=(left ventricle)) OR TS=(LV dysfunction)) OR TS=(left ventricular dysfunction)) OR TS=(LVMI)) OR TS=(ejection fraction)	182315
#2	TS=((pre-diabetes) OR (non-diabetic) OR (insulin resistance) OR (prediabetes))	139675
#3	TS=((randomized clinical trial) OR (randomized controlled trial) OR (rct))	518339
#4	TS=((metformin) OR (biguanide) OR (glucophage))	27202
#5	((((TS=((metformin) OR (biguanide) OR (glucophage))) AND TS=((pre-diabetes) OR (non-diabetic) OR (insulin resistance) OR (prediabetes))) AND TS=((randomized clinical trial) OR (randomized controlled trial) OR (rct)))) AND TS=(left ventricle OR LV dysfunction OR LVMI OR left ventricular dysfunction OR ejection fraction)	8
**medRxiv (5th of November)**		
#1	for term "Metformin AND randomized AND (LVMI OR LVEF OR BNP OR Heart failure)" and abstract or title "Metformin AND randomized AND (LVMI OR LVEF OR BNP OR Heart failure)" (match all words)	**2**
**Total**		**281**

2. Reasons for Excluding Abstracts

Table S2: Reasons for Excluding abstracts

Reason	Cochrane, Medline, Scopus, PubMed	ClinicalTrials.gov	Total
All patients on metformin	1		1
Animal model	3		3
Cohort	13		13
Conference	3		3
Cross-sectional	5		5
Diabetic patients	24	8	33
Invitro model	1		1
No CVS outcome	2	63	65
Other intervention	19	13	32
Protocol	11		11
Review	74		74
Published		3	3
Terminated		2	2
	156	89	245

3. Reasons for Excluding Full-Text Research

Table S3: Included and excluded full Text research with reasons

Text	Reason
Stakos, D. A., Schuster, D. P., Sparks, E. A., Wooley, C. F., Osei, K., & Boudoulas, H. (2005). Long term cardiovascular effects of oral antidiabetic agents in non-diabetic patients with insulin resistance: double blind, prospective, randomised study. Heart, 91(5), 589–594. https://doi.org/10.1136/hrt.2003.027722	
Cadeddu, C., Nocco, S., Cugusi, L., Deidda, M., Fabio, O., Bandino, S., … Mercuro, G. (2016). Effects of Metformin and Exercise Training, Alone or in Combination, on Cardiac Function in Individuals with Insulin Resistance. *Cardiol Ther*, *5*(1), 63–73. https://doi.org/10.1007/s40119-016-0057-3	Non-randomized
Gorter, T. M., Lexis, C. P. H., Lipsic, E., Van Veldhuisen, D. J., Van Melle, J. P., Willems, T. P., … Van Der Harst, P. (2015). Right ventricular dysfunction after acute myocardial infarction in the era of percutaneous coronary intervention: a substudy of the {GIPS-III} randomized clinical trial. *Eur. J. Heart Fail.*, *17*, 227-228. https://doi.org/10.1002/ejhf.277	Part of another study
Eppinga, R. N., Kofink, D., Dullaart, R. P. F., Dalmeijer, G. W., Lipsic, E., Van Veldhuisen, D. J., … Van Der Harst, P. (2017). Effect of Metformin on Metabolites and Relation with Myocardial Infarct Size and Left Ventricular Ejection Fraction after Myocardial Infarction. *Circ. Cardiovasc. Genet.*, *10*(1). https://doi.org/10.1161/CIRCGENETICS.116.001564	Part of another study
Kurmanbekova, B., Noruizbaeva, A., Osmankulova, G., & Rustambekova, A. (2021). Evaluation of the beneficial effect of metformin on clinical indicators of heart failure patients with coronary artery disease and impaired glucose tolerance within a 12-month follow-up. *Am. Heart J.*, *242*, 164. https://doi.org/10.1016/j.ahj.2021.10.047	
Al Ali, L., Hartman, M. T., Lexis, C. P. H., Hummel, Y. M., Lipsic, E., van Melle, J. P., … van der Harst, P. (2016). The effect of metformin on diastolic function in patients presenting with st-elevation myocardial infarction. *PLoS ONE*, *11*(12), e0168340. https://doi.org/10.1371/journal.pone.0168340	
Eppinga, R. N., Hartman, M. H. T., van Veldhuisen, D. J., Lexis, C. P. H., Connelly, M. A., Lipsic, E., … Dullaart, R. P. F. (2016). Effect of Metformin Treatment on Lipoprotein Subfractions in {Non-Diabetic} Patients with Acute Myocardial Infarction: A Glycometabolic Intervention as Adjunct to Primary Coronary Intervention in {ST} Elevation Myocardial Infarction ({GIPS-III}) Trial. *PLoS One*, *11*(1). https://doi.org/10.1371/journal.pone.0145719	Part of another study
Sardu, C., Pieretti, G., D'Onofrio, N., Ciccarelli, F., Paolisso, P., Passavanti, M. B., … Barbieri, M. (2018). Inflammatory Cytokines and SIRT1 Levels in Subcutaneous Abdominal Fat: Relationship With Cardiac Performance in Overweight Pre-diabetics Patients. *Frontiers in Physiology*, *9*(AUG). https://doi.org/10.3389/fphys.2018.01030	Part of another study
Cannon, M. V, Lexis, C. P., Rogier Van Der Velde, A., Van Der Horst, I. C., Lipsic, E., Dokter, M. M., … De Boer, R. A. (2014). Metformin lowers circulating adiponectin levels in patients with myocardial infarction. *Circulation*, *130*.	No CVS outcomes
Ladeiras-Lopes, R., Sampaio, F., Leite, S., Santos-Ferreira, D., Vilela, E., Leite-Moreira, A., … Fontes-Carvalho, R. (2021). Metformin in non-diabetic patients with metabolic syndrome and diastolic dysfunction: the MET-DIME randomized trial. Endocrine, 72(3), 699–710. https://doi.org/10.1007/s12020-021-02687-0	

EUCTR2017-004149-26-DE. (2018). Effect of Metformin in insulin resistant patients with heart failure with reduced ejection fraction. *Https://Trialsearch.Who.Int/Trial2.Aspx?TrialID=EUCTR2017-004149-26-DE*.	Protocol
Mohan, M., Mcswiggan, S., Baig, F., Rutherford, L., & Lang, C. C. (2015). Metformin and its effects on myocardial dimension and left ventricular hypertrophy in normotensive patients with coronary heart disease (The MET-REMODEL Study): Rationale and design of the MET-REMODEL study. *Cardiovascular Therapeutics*, *33*(1), 1–8. https://doi.org/10.1111/1755-5922.12101	Part of another study
Eppinga, R. N., Hartman, M. H. T., Van Veldhuisen, D. J., Lexis, C. P. H., Connelly, M. A., Lipsic, E., … Dullaart, R. P. F. (2016). Effect of metformin treatment on lipoprotein subfractions in non-diabetic patients with acute myocardial infarction: A glycometabolic intervention as adjunct to primary coronary intervention in st elevation myocardial infarction (GIPS-III) trial. *PLoS ONE*, *11*(1), e0145719. https://doi.org/10.1371/journal.pone.0145719	Part of another study
Lexis, C. P. H. H., Van Der Horst, I. C. C. C., Lipsic, E., van der Harst, P., Van Der Horst-Schrivers, A. N. A. A., Wolffenbuttel, B. H. R. R., … De Smet, B. J. G. L. G. L. (2012). Metformin in non-diabetic patients presenting with st elevation myocardial infarction: Rationale and design of the glycometabolic intervention as adjunct to primary percutaneous intervention in st elevation myocardial infarction (gips)-iii trial. *Cardiovascular Drugs and Therapy*, *26*(5), 417–426. https://doi.org/10.1007/s10557-012-6413-1	Part of another study
Velázquez, H., Meaney, A., Galeana, C., Zempoalteca, J. C., Izaguirre-Gutiérrez, F., Nájera, N., … Meaney, E. (2016). Metformin enhances left ventricular function in patients with metabolic syndrome. *Revista Mexicana de Cardiologia*, *27*(1), 16–25.	
Mohan, M., Al-Talabany, S., McKinnie, A., Mordi, I. R., Singh, J. S. S. S., Gandy, S. J., … Lang, C. C. (2019). A randomized controlled trial of metformin on left ventricular hypertrophy in patients with coronary artery disease without diabetes: The MET-REMODEL trial. *European Heart Journal*, *40*(41), 3409–3417. https://doi.org/10.1093/eurheartj/ehz203	
Sardu, C., Trotta, M. C., Pieretti, G., Gatta, G., Ferraro, G., Nicoletti, G. F., … Marfella, R. (2021). MicroRNAs modulation and clinical outcomes at 1 year of follow-up in obese patients with pre-diabetes treated with metformin vs. placebo. *Acta Diabetologica*, *58*(10), 1381–1393. https://doi.org/10.1007/s00592-021-01743-5	
Wong, A. K. F., Symon, R., AlZadjali, M. A., Ang, D. S. C., Ogston, S., Choy, A., … Lang, C. C. (2012). The effect of metformin on insulin resistance and exercise parameters in patients with heart failure. *Eur. J. Heart Fail.*, *14*(11), 1303–1310. https://doi.org/10.1093/eurjhf/hfs106	
Larsen, A. H., Jessen, N., Nørrelund, H., Tolbod, L. P., Harms, H. J., Feddersen, S. S., … Wiggers, H. (2019). A randomised, double-blind, placebo-controlled trial of metformin on myocardial efficiency in insulin-resistant chronic heart failure patients without diabetes. *Eur. J. Heart Fail.*, *22*(9), 1628–1637. https://doi.org/10.1002/ejhf.1656	

4. Calculation Formulas

Table S4: Calculation formulas

4.1. Formula for calculating correlation coefficient using pre-test, post-test, and change values<br>$$Corr_E = \frac{SD^2_{E,baseline} + SD^2_{E,final} - SD^2_{E,change}}{2 \times SD_{E,baseline} \times SD_{E,final}}$$
4.2. Formula for standard deviation for change from pre-test and post-test data<br>$$SD_{E,change} = \sqrt{SD^2_{E,baseline} + SD^2_{E,final} - (2 \times Corr \times SD_{E,baseline} \times SD_{E,final})}$$
4.3. Formula for standard deviation using mean difference and 95% CI in the change between groups<br>$SE = (\text{upper limit} - \text{lower limit})/3.92$<br>The 3.92 was replaced by the t-value for a 95% confidence interval from a comparison of a sample size of x with a sample size of y at (x + y – 2) degrees of freedom.<br>$$SD = \frac{SE}{\sqrt{\frac{1}{N_E} + \frac{1}{N_C}}}$$<br>The standard deviation is the average of the standard deviations of the experimental and control arms and should be entered into RevMan twice (once for each intervention group).

5. Estimated Correlation Coefficients and Standard Deviations

Table S5a: Estimation of correlation coefficients for LVMI based on results of published studies

SD Pre	SD Post	SD LVMI T-change	Group	r	Study
25	22	8.86	T	0.9368185455	Larsen 2020
25	29	8.86	C	0.9568968276	Larsen 2020
10.9	9.2	7.8	T	0.7110590347	EMPA-HEART
12.8	11.7	5.7	C	0.8955662393	EMPA-HEART
19.8	22.6	6.6	C	0.9600876017	https://doi.org/10.1016/j.jacc.2020.11.008
19	17.1	10.2	C	0.8454447522	IDDIA trial
54.4	60.1	13.4	C	0.9775083806	EMPA-TROPISM
				0.693	Doi: 10.1038/hr.2013.64
22.2	18.66	16.96	T	0.6679436671	Ali 2015
20.7	17.33	16.7	C	0.6271118192	Ali 2015

r: Pearson’s correlation coefficient
T: Metformin; C: Placebo or SOC

Table S5b. Correlation coefficients for LVEF based on two of the included studies

Pre	Post	Change	Group	r	Study
9	10	7.82	T	0.66582	Larsen 2020
6	11	7.82	C	0.7261181818	Larsen 2020
8.15	7.41	7.63	C	0.5225379399	Ali 2015
10.37	8.89	7.78	T	0.6835966864	Ali 2015

r: Pearson’s correlation coefficient
T: Metformin; C: Placebo or SOC
The correlation coefficient was reported directly in one study and calculated in the remaining studies using the following formula:

$$\mathrm{Corr}_E = \frac{\mathrm{SD}^2_{E,\mathrm{baseline}} + \mathrm{SD}^2_{E,\mathrm{final}} - \mathrm{SD}^2_{E,\mathrm{change}}}{2 \times SD_{E,\mathrm{baseline}} \times SD_{E,\mathrm{final}}}$$

Table S5c: Estimated standard deviation for Larsen's 2020 study

LLCI	ULCI	SE	n1	pooled SD	t-score	t-score x 2	SD group	Study	Parameter
-4.30	6.30	2.61	36.00	15.65	**2.03**	4.06	7.82	Larsen 2020	LVEF
-10.00	2.00	2.95	36.00	17.71	**2.03**	4.06	8.86	Larsen 2020	LVMI
-2.00	5.00	1.72	36.00	10.33	**2.03**	4.06	5.17	Larsen 2020	Ee ratio
-10.80	11.10	5.39	36.00	32.33	**2.03**	4.06	16.16	Larsen 2020	EDV
-7.00	8.00	3.69	36.00	22.14	**2.03**	4.06	11.07	Larsen 2020	QoL

6. Leave One-Out Sensitivity Analysis For LVMI

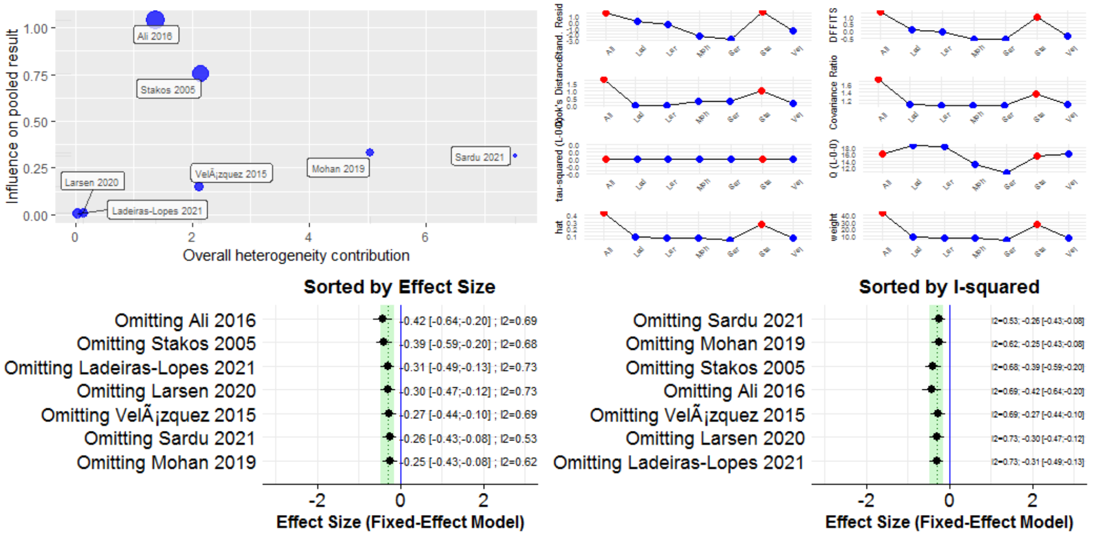

Figure S1: Sensitivity analysis for the effect of metformin on LVMI using SMCC as the unit of analysis

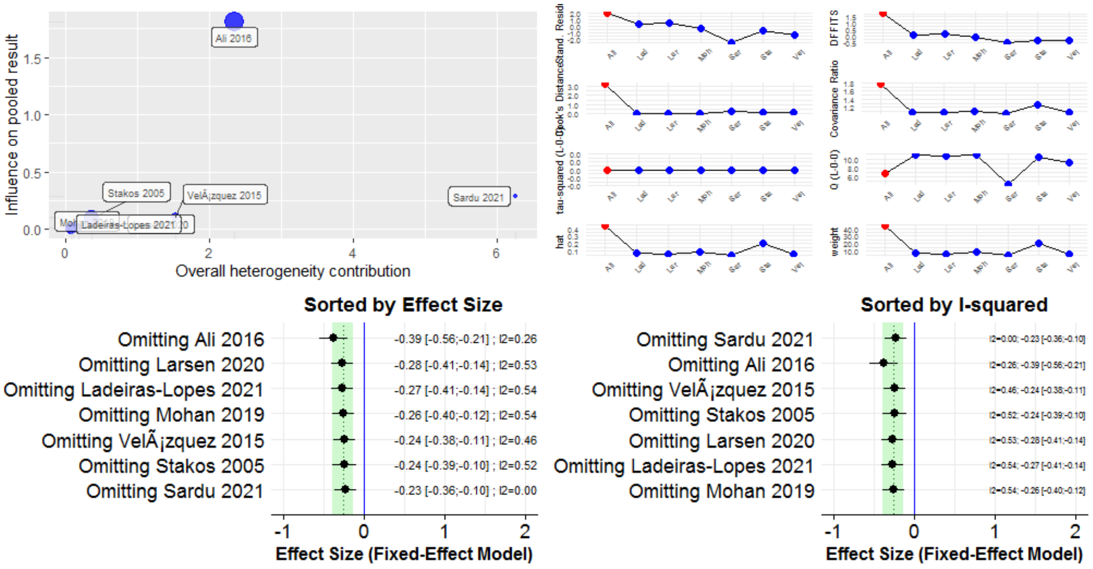

Figure S2: Sensitivity analysis for the effect of metformin on LVMI using SMCR as the unit of analysis

7. Funnel Plot Asymmetry for The Effect Of Metformin On LVMI

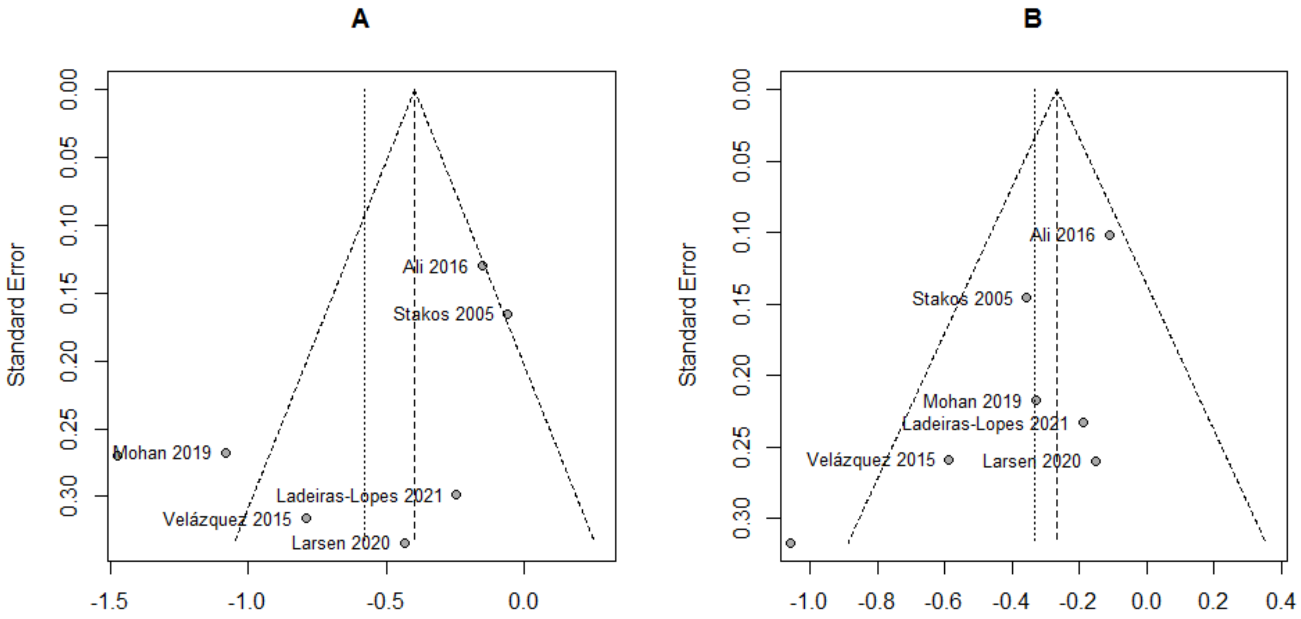

Figure S3. Funnel plots for LVMI using (A) SMCC (B) SMCR

8. Leave One-Out Sensitivity Analysis for The Effect Of Metformin On LVEF

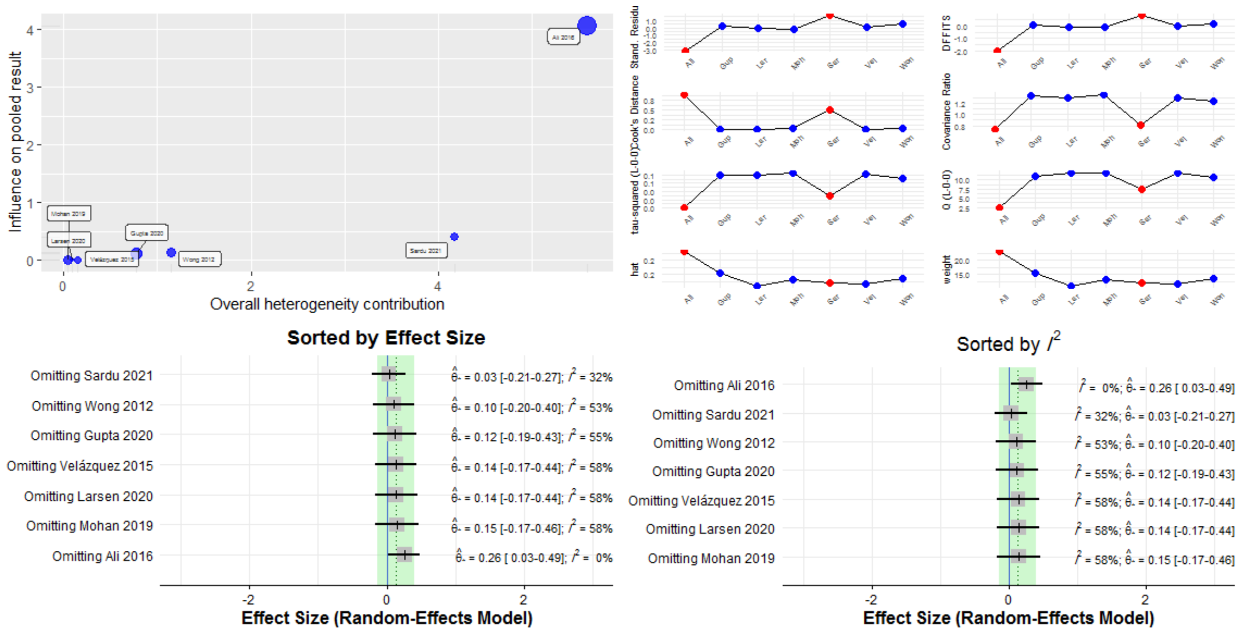

Figure S4: Sensitivity analysis for the effect of metformin on LVEF using SMCC as the unit of analysis

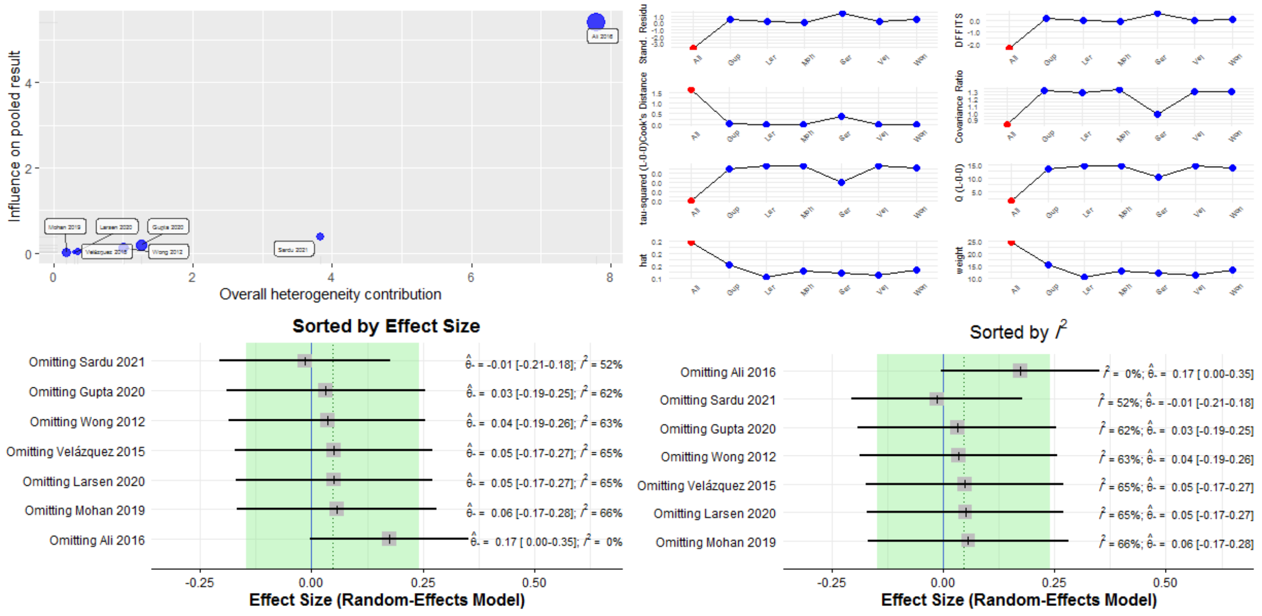

Figure S5: Sensitivity analysis for the effect of metformin on LVEF using SMCR as the unit of analysis

9. Funnel Plot Asymmetry for The Effect of Metformin On LVEF

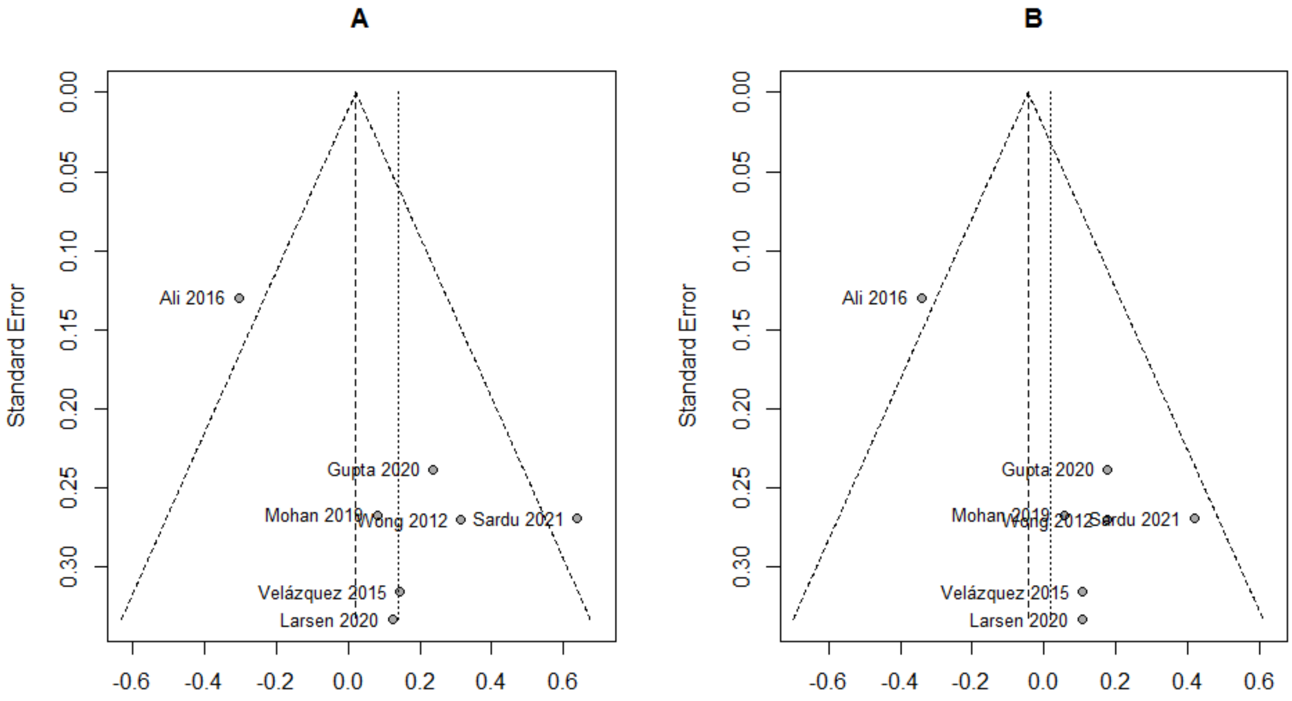

Figure S6. Funnel plots for LVEF using (A) SMCC (B) SMCR

10. Sensitivity Analysis for The Effect Of Metformin On E/E' Ratio

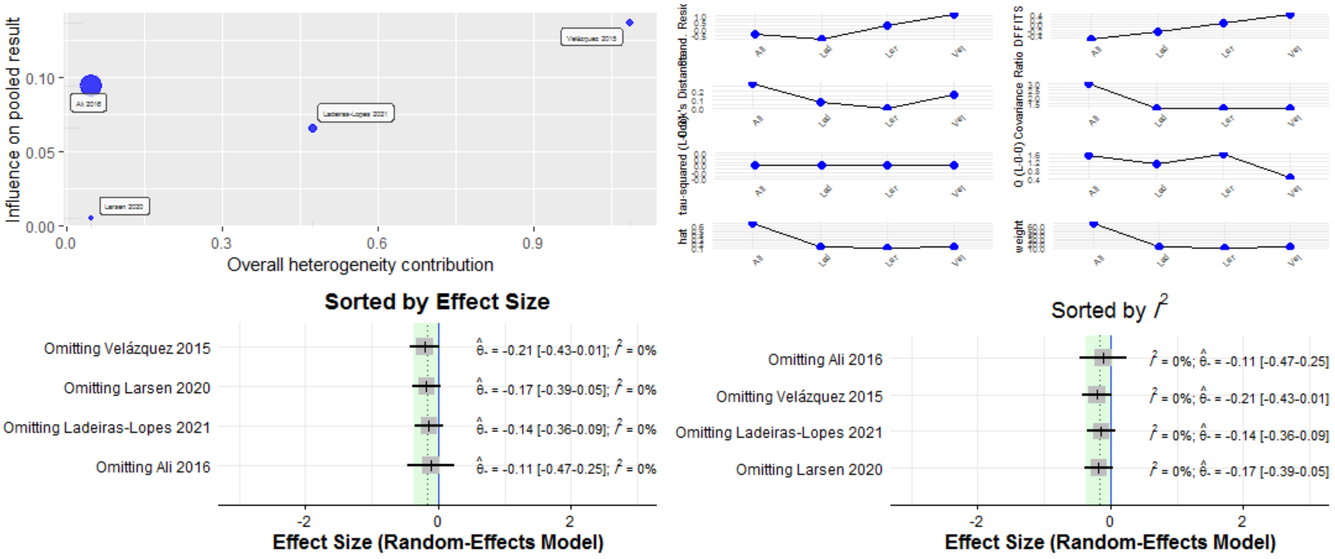

Figure S7: Sensitivity analysis for the effect of metformin on E/e' ratio using SMCC as the unit of analysis

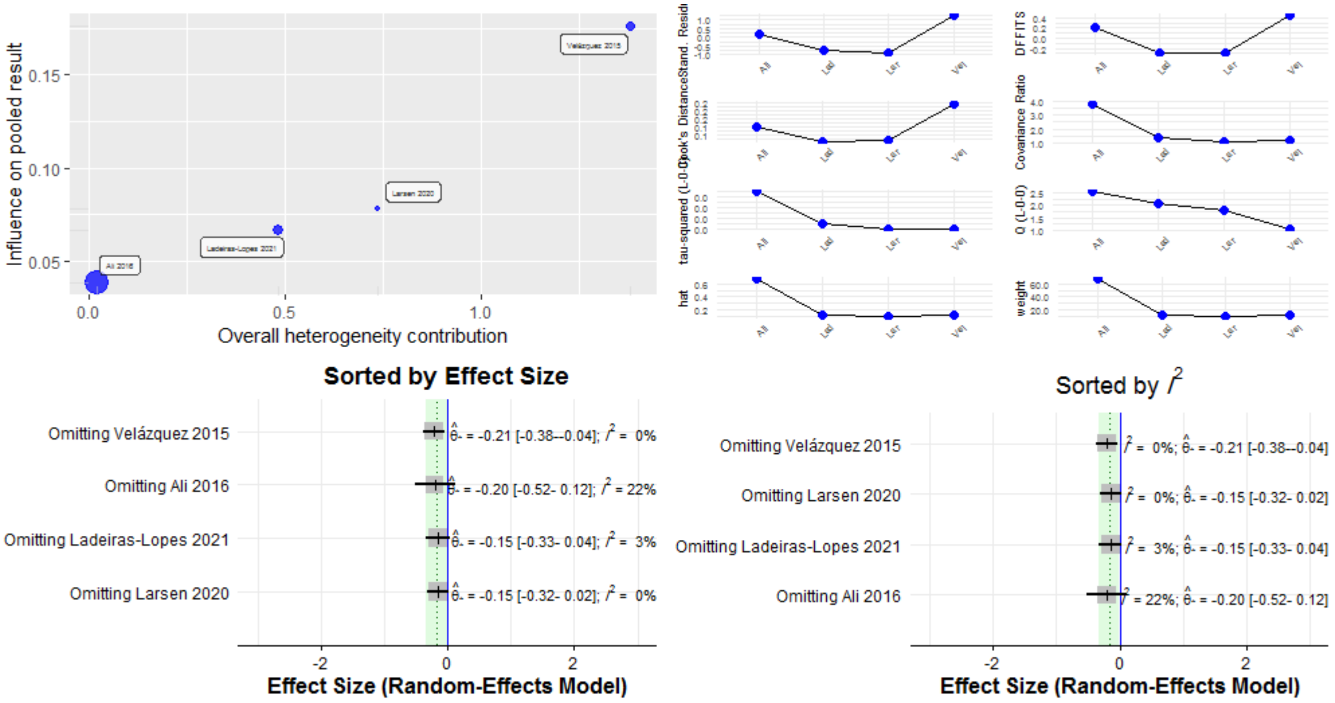

Figure S8: Sensitivity analysis for the effect of metformin on E/e' ratio using SMCR as the unit of analysis

11. Funnel Plot Asymmetry for The Effect Of Metformin On E/E' Ratio

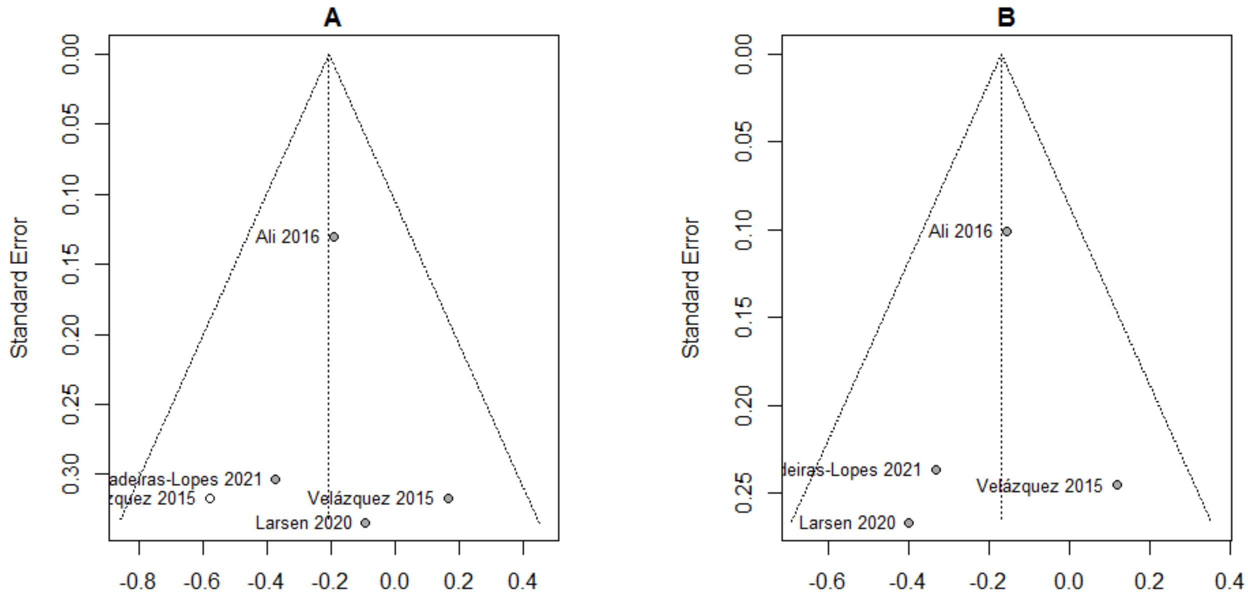

Figure S9a: Funnel plots for E/e' ratio using (A) SMCC (B) SMCR

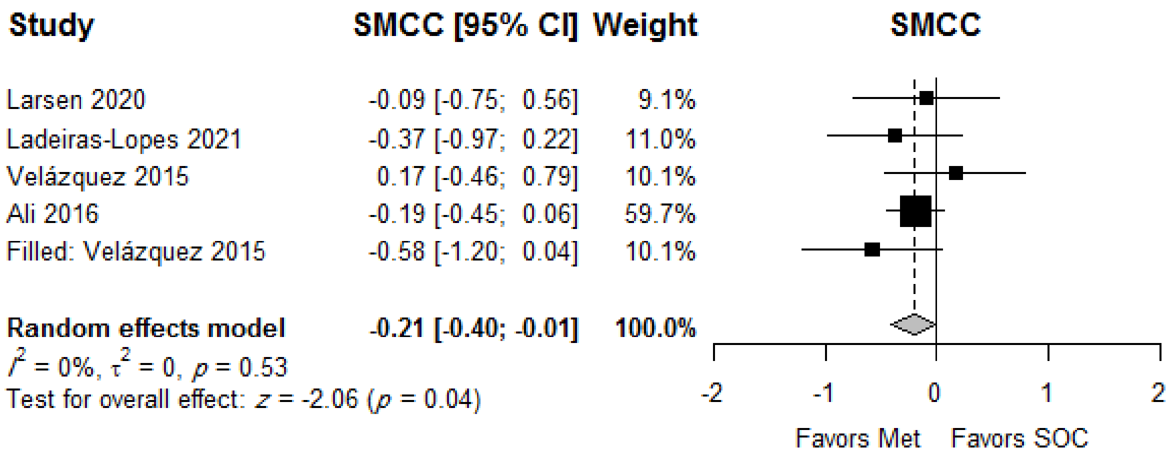

Figure S9b: Pooled SMCC for the effect of metformin on E/e' ratio after using trim-fill method

12. Sensitivity analysis for the effect of metformin on NT-ProBNP/BNP

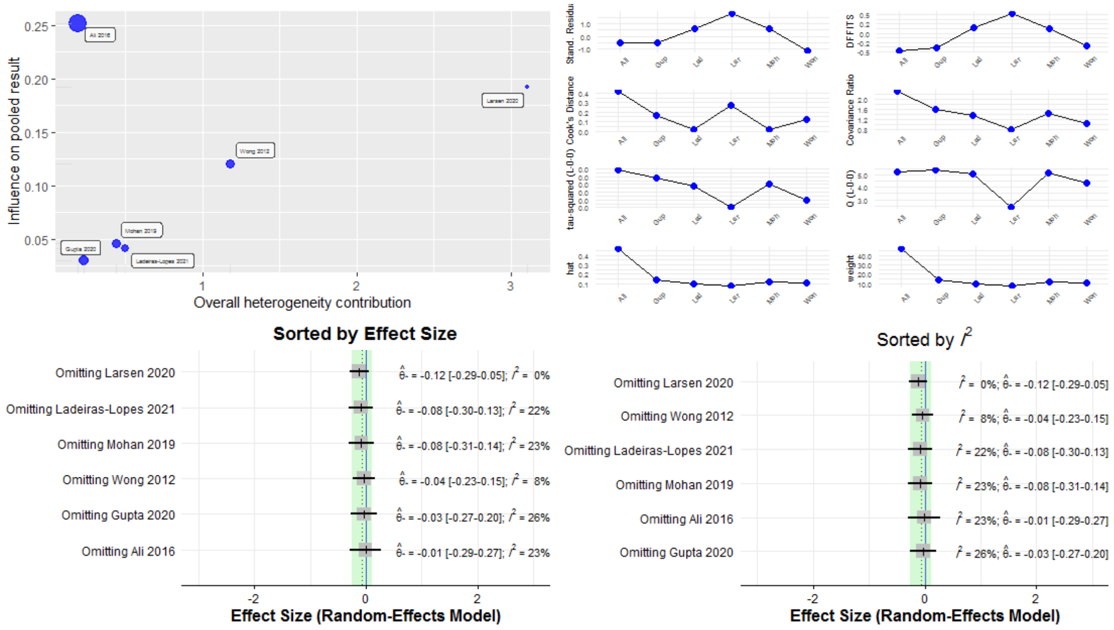

Figure S10: Sensitivity analysis for the effect of metformin on BNP/NT-ProBNP using SMCC as the unit of analysis

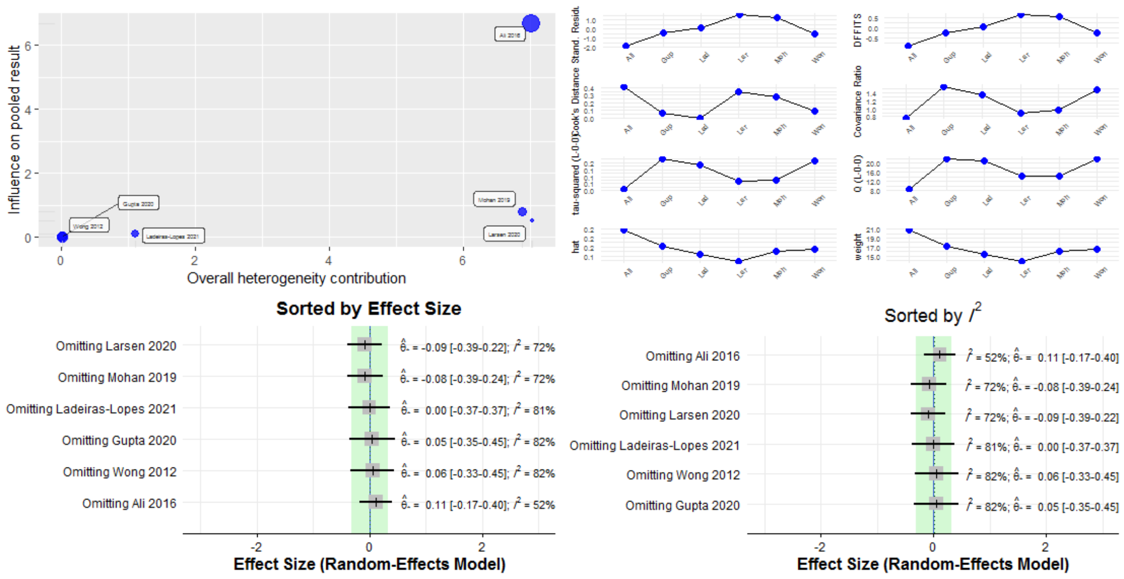

Figure S11: Sensitivity analysis for the effect of metformin on BNP/NT-ProBNP using SMCR as the unit of analysis

13. Funnel plot asymmetry for the effect of metformin on NT-ProBNP/BNP

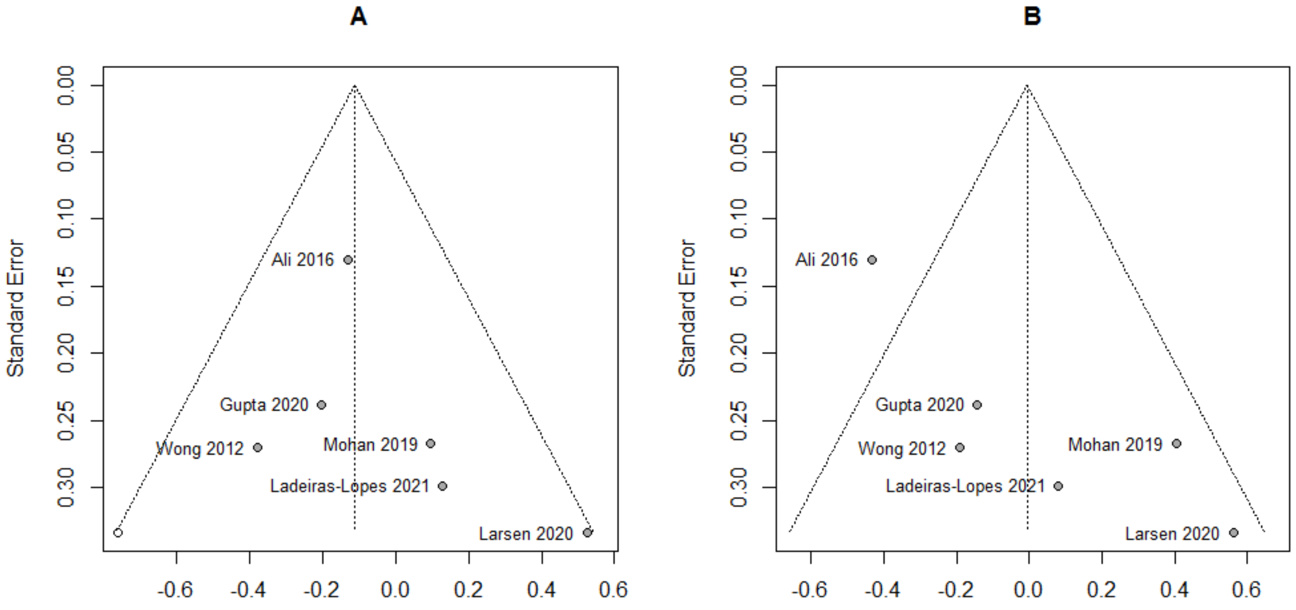

Figure S12: Funnel plots for BNP/NT-ProBNP ratio using (A) SMCC (B) SMCR
