# Supplementary material for: Effect of metformin on left ventricular mass and functional parameters in non-diabetic patients: a meta-analysis of randomized clinical trials
Source: BMC Cardiovasc Disord. 2022 Sep 10;22:405. doi: 10.1186/s12872-022-02845-w (PMC9464374; doi:10.1186/s12872-022-02845-w)
Supplement: Supplementary file 2 — Additional file 2. Risk of Bias Assessment in the Effect ofMetformin on Left Ventricular Mass Index (LVMI). [file 12872_2022_2845_MOESM2_ESM.pdf]

Effect of Metformin on Left Ventricular Mass and Functional Parameters in Non-Diabetic Patients: A Meta -analysis of Randomized Clinical Trials

Ahmed M. Kamel, MSc [a], Nirmeen Sabry, PhD [a], Samar Farid, PhD [a]

[a] *Clinical Pharmacy Department, Faculty of Pharmacy Cairo University, Cairo, 11562, Egypt*

***Corresponding author**

Ahmed Mohamed Kamel, MSc

Cairo University, College of Pharmacy, Department of Clinical Pharmacy, Egypt.

E-mail addresses: ahmedm.kamel@pharma.cu.edu.eg

Tel: +010-0676-6275

Fax: +011-202-25320005

ORCID ID:

Ahmed M. Kamel: 0000-0002-3791-5998

Nirmeen Sabry: 0000-0003-0478-0772

Samar Farid: 0000-0002-6048-847X

Appendix 2: Risk of Bias assessment in the effect of Metformin on Left Ventricular Mass Index (LVMI)

<table>
<tr><td>Unique ID</td><td>LVMI-Stakos</td><td>Study ID</td><td>Stakos 2005</td><td>Assessor</td><td>Ahmed</td></tr>
<tr><td>Ref or Label</td><td>1</td><td>Aim</td><td>adhering to intervention (the 'per-protocol' effect)</td><td>The effect of adhering to intervention…</td><td>occurance of non-protocol interventions; failures in implementing the intervention that could have affected the outcome; non-adherence to their assigned intervention by trial participants</td></tr>
<tr><td>Experimental</td><td>Metformin</td><td>Comparator</td><td>Placebo</td><td>Source</td><td>Journal article(s)</td></tr>
<tr><td>Outcome</td><td>LVMI</td><td>Results</td><td></td><td>Weight</td><td>1</td></tr>
<tr><th>Domain</th><th colspan="3">Signalling question</th><th>Response</th><th>Comments</th></tr>
<tr><td rowspan="4">Bias arising from the randomization process</td><td colspan="3">1.1 Was the allocation sequence random?</td><td>NI</td><td rowspan="2">The shape of active drug tablets (glipizide and metformin) is different. For this reason for each active drug we created an identical placebo</td></tr>
<tr><td colspan="3">1.2 Was the allocation sequence concealed until participants were enrolled and assigned to interventions?</td><td>NI</td></tr>
<tr><td colspan="3">1.3 Did baseline differences between intervention groups suggest a problem with the randomization process?</td><td>N</td><td></td></tr>
<tr><td colspan="3">Risk of bias judgement</td><td>Some concerns</td><td></td></tr>
<tr><td rowspan="7">Bias due to deviations from intended interventions</td><td colspan="3">2.1 Were participants aware of their assigned intervention during the trial?</td><td>PN</td><td rowspan="2">Randomized DB</td></tr>
<tr><td colspan="3">2.2 Were carers and people delivering the interventions aware of participants' assigned intervention during the trial?</td><td>PN</td></tr>
<tr><td colspan="3">2.3. [If applicable:] If Y/PY/NI to 2.1 or 2.2: Were important non-protocol interventions balanced across intervention groups?</td><td></td><td></td></tr>
<tr><td colspan="3">2.4. [If applicable:] Were there failures in implementing the intervention that could have affected the outcome?</td><td></td><td></td></tr>
<tr><td colspan="3">2.5. [If applicable:] Was there non-adherence to the assigned intervention regimen that could have affected participants' outcomes?</td><td></td><td></td></tr>
<tr><td colspan="3">2.6. If N/PN/NI to 2.3, or Y/PY/NI to 2.4 or 2.5: Was an appropriate analysis used to estimate the effect of adhering to the intervention?</td><td>NA</td><td></td></tr>
<tr><td colspan="3">Risk of bias judgement</td><td>Some concerns</td><td></td></tr>
<tr><td rowspan="5">Bias due to missing outcome data</td><td colspan="3">3.1 Were data for this outcome available for all, or nearly all, participants randomized?</td><td>N</td><td>Data was missing for ~13% of the patients</td></tr>
<tr><td colspan="3">3.2 If N/PN/NI to 3.1: Is there evidence that result was not biased by missing outcome data?</td><td>N</td><td></td></tr>
<tr><td colspan="3">3.3 If N/PN to 3.2: Could missingness in the outcome depend on its true value?</td><td>NI</td><td rowspan="2"></td></tr>
<tr><td colspan="3">3.4 If Y/PY/NI to 3.3: Is it likely that missingness in the outcome depended on its true value?</td><td>PN</td></tr>
<tr><td colspan="3">Risk of bias judgement</td><td>Some concerns</td><td></td></tr>
<tr><td rowspan="6">Bias in measurement of the outcome</td><td colspan="3">4.1 Was the method of measuring the outcome inappropriate?</td><td>N</td><td>ANOVA was used</td></tr>
<tr><td colspan="3">4.2 Could measurement or ascertainment of the outcome have differed between intervention groups?</td><td>N</td><td>Two time periods for the control groups. However, it is the same device</td></tr>
<tr><td colspan="3">4.3 Were outcome assessors aware of the intervention received by study participants?</td><td>PN</td><td></td></tr>
<tr><td colspan="3">4.4 If Y/PY/NI to 4.3: Could assessment of the outcome have been influenced by knowledge of intervention received?</td><td>NA</td><td rowspan="2"></td></tr>
<tr><td colspan="3">4.5 If Y/PY/NI to 4.4: Is it likely that assessment of the outcome was influenced by knowledge of intervention received?</td><td>NA</td></tr>
<tr><td colspan="3">Risk of bias judgement</td><td>Low</td><td></td></tr>
<tr><td rowspan="4">Bias in selection of the reported result</td><td colspan="3">5.1 Were the data that produced this result analysed in accordance with a pre-specified analysis plan that was finalized before unblinded outcome data were available for analysis?</td><td>NI</td><td>No pre-registered protocol</td></tr>
<tr><td colspan="3">5.2 ... multiple eligible outcome measurements (e.g. scales, definitions, time points) within the outcome domain?</td><td>N</td><td></td></tr>
<tr><td colspan="3">5.3 ... multiple eligible analyses of the data?</td><td>N</td><td>Only per-protocol reported so</td></tr>
<tr><td colspan="3">Risk of bias judgement</td><td>Some concerns</td><td></td></tr>
<tr><td>Overall bias</td><td colspan="3">Risk of bias judgement</td><td>Some concerns</td><td></td></tr>
</table>

**Unique ID**	LVMI-Mohan	**Study ID**	Mohan 2019	**Assessor**	Ahmed
**Ref or Label**	2	**Aim**	adhering to intervention (the 'per-protocol' effect)	**The effect of adhering to intervention…**	occurance of non-protocol interventions; failures in implementing the intervention that could have affected the outcome; non-adherence to their assigned intervention by trial participants
**Experimental**	Metformin	**Comparator**	Placebo	**Source**	Journal article(s)
**Outcome**	LVMI	**Results**		**Weight**	1

Domain	Signalling question	Response	Comments
**Bias arising from the randomization process**	1.1 Was the allocation sequence random?	Y	Randomization was carried out by Tayside Pharmaceuticals using a validated block The investigating team did not have access to the key until after analysis had taken place
	1.2 Was the allocation sequence concealed until participants were enrolled and assigned to interventions?	Y	
	1.3 Did baseline differences between intervention groups suggest a problem with the randomization process?	N	
	**Risk of bias judgement**	**Low**	
**Bias due to deviations from intended interventions**	2.1 Were participants aware of their assigned intervention during the trial?	N	DB-RCT The investigating team did not have access to the key until after analysis had taken place
	2.2 Were carers and people delivering the interventions aware of participants' assigned intervention during the trial?	N	
	2.3. [If applicable:] If Y/PY/NI to 2.1 or 2.2: Were important non-protocol interventions balanced across intervention groups?	NA	
	2.4. [If applicable:] Were there failures in implementing the intervention that could have affected the outcome?	PN	Drop out rates were similar across groups
	2.5. [If applicable:] Was there non-adherence to the assigned intervention regimen that could have affected participants' outcomes?	PN	Drop out rates were similar across groups
	2.6. If N/PN/NI to 2.3, or Y/PY/NI to 2.4 or 2.5: Was an appropriate analysis used to estimate the effect of adhering to the intervention?	NA	
	**Risk of bias judgement**	**Low**	
**Bias due to missing outcome data**	3.1 Were data for this outcome available for all, or nearly all, participants randomized?	Y	Data analyzed using miTT and per-protocol and similar results were found
	3.2 If N/PN/NI to 3.1: Is there evidence that result was not biased by missing outcome data?	NA	
	3.3 If N/PN to 3.2: Could missingness in the outcome depend on its true value?	NA	
	3.4 If Y/PY/NI to 3.3: Is it likely that missingness in the outcome depended on its true value?	NA	
	**Risk of bias judgement**	**Low**	
**Bias in measurement of the outcome**	4.1 Was the method of measuring the outcome inappropriate?	N	DB-RCT and the protocol and method of assessment were pre-specified
	4.2 Could measurement or ascertainment of the outcome have differed between intervention groups?	PN	
	4.3 Were outcome assessors aware of the intervention received by study participants?	N	
	4.4 If Y/PY/NI to 4.3: Could assessment of the outcome have been influenced by knowledge of intervention received?	NA	
	4.5 If Y/PY/NI to 4.4: Is it likely that assessment of the outcome was influenced by knowledge of intervention received?	NA	
	**Risk of bias judgement**	**Low**	
**Bias in selection of the reported result**	5.1 Were the data that produced this result analysed in accordance with a pre-specified analysis plan that was finalized before unblinded outcome data were available for analysis?	Y	The protocol and method of analysis were published and no deviations occured
	5.2 ... multiple eligible outcome measurements (e.g. scales, definitions, time points) within the outcome domain?	N	Change from baseline was defined a priori
	5.3 ... multiple eligible analyses of the data?	N	Change from baseline only
	**Risk of bias judgement**	**Low**	
**Overall bias**	**Risk of bias judgement**	**Low**	

**Unique ID**	LVMI-Larsen	**Study ID**	LVMI-Larsen	**Assessor**	Ahmed
**Ref or Label**	3	**Aim**	adhering to intervention (the 'per-protocol' effect)	**The effect of adhering to intervention…**	occurance of non-protocol interventions; failures in implementing the intervention that could have affected the outcome; non-adherence to their assigned intervention by trial participants
**Experimental**	Metformin	**Comparator**	Placebo	**Source**	Journal article(s)
**Outcome**	LVMI	**Results**		**Weight**	1

Domain	Signalling question	Response	Comments
**Bias arising from the randomization process**	1.1 Was the allocation sequence random?	Y	The unequal treatment assignment (19vs. 17 patients) was due to a pre-established computer-generated sequence equally balanced at 40
	1.2 Was the allocation sequence concealed until participants were enrolled and assigned to interventions?	PY	
	1.3 Did baseline differences between intervention groups suggest a problem with the randomization process?	N	
	**Risk of bias judgement**	**Low**	
**Bias due to deviations from intended interventions**	2.1 Were participants aware of their assigned intervention during the trial?	PN	
	2.2 Were carers and people delivering the interventions aware of participants' assigned intervention during the trial?	PN	
	2.3. [If applicable:] If Y/PY/NI to 2.1 or 2.2: Were important non-protocol interventions balanced across intervention groups?	PN	Dropout rates were similar
	2.4. [If applicable:] Were there failures in implementing the intervention that could have affected the outcome?	PN	Dropout rates were similar
	2.5. [If applicable:] Was there non-adherence to the assigned intervention regimen that could have affected participants' outcomes?	N	Pill count showed good compliance
	2.6. If N/PN/NI to 2.3, or Y/PY/NI to 2.4 or 2.5: Was an appropriate analysis used to estimate the effect of adhering to the intervention?	Y	ITT used
	**Risk of bias judgement**	**Low**	
**Bias due to missing outcome data**	3.1 Were data for this outcome available for all, or nearly all, participants randomized?	Y	All 36 patients initially randomized were analysed
	3.2 If N/PN/NI to 3.1: Is there evidence that result was not biased by missing outcome data?	NA	
	3.3 If N/PN to 3.2: Could missingness in the outcome depend on its true value?	NA	
	3.4 If Y/PY/NI to 3.3: Is it likely that missingness in the outcome depended on its true value?	NA	
	**Risk of bias judgement**	**Low**	
**Bias in measurement of the outcome**	4.1 Was the method of measuring the outcome inappropriate?	N	
	4.2 Could measurement or ascertainment of the outcome have differed between intervention groups?	PN	DB-RCT
	4.3 Were outcome assessors aware of the intervention received by study participants?	PN	DB-RCT
	4.4 If Y/PY/NI to 4.3: Could assessment of the outcome have been influenced by knowledge of intervention received?	NA	
	4.5 If Y/PY/NI to 4.4: Is it likely that assessment of the outcome was influenced by knowledge of intervention received?	NA	
	**Risk of bias judgement**	**Low**	
**Bias in selection of the reported result**	5.1 Were the data that produced this result analysed in accordance with a pre-specified analysis plan that was finalized before unblinded outcome data were available for analysis?	PY	a total of 36 patients were required to detect a relative WMI difference of 0.6 mL·mmHg·m−2·106 between the two treatment groups (a 2-sided ?? of 0.05 at 80% power) while allowing for 17% dropout. Data
	5.2 ... multiple eligible outcome measurements (e.g. scales, definitions, time points) within the outcome domain?	N	
	5.3 ... multiple eligible analyses of the data?	N	
	**Risk of bias judgement**	**Low**	
**Overall bias**	**Risk of bias judgement**	**Low**	

Unique ID	LVMI-Ladeiras-Lopes	Study ID	Ladeiras-Lopes 2021	Assessor	Ahmed
Ref or Label	4	Aim	adhering to intervention (the 'per-protocol' effect)	The effect of adhering to intervention…	occurance of non-protocol interventions; failures in implementing the intervention that could have affected the outcome; non-adherence to their assigned intervention by trial participants
Experimental	Metformin	Comparator	SOC	Source	Journal article(s)
Outcome	LVMI	Results		Weight	1

Domain	Signalling question	Response	Comments
Bias arising from the randomization process	1.1 Was the allocation sequence random?	PY	Minimization technique used which is complex and can't be deduced
	1.2 Was the allocation sequence concealed until participants were enrolled and assigned to interventions?	PY	
	1.3 Did baseline differences between intervention groups suggest a problem with the randomization process?	N	
	**Risk of bias judgement**	**Low**	
Bias due to deviations from intended interventions	2.1 Were participants aware of their assigned intervention during the trial?	Y	Open label
	2.2 Were carers and people delivering the interventions aware of participants' assigned intervention during the trial?	Y	
	2.3. [If applicable:] If Y/PY/NI to 2.1 or 2.2: Were important non-protocol interventions balanced across intervention groups?	PY	Life style modifications was in both
	2.4. [If applicable:] Were there failures in implementing the intervention that could have affected the outcome?	PN	
	2.5. [If applicable:] Was there non-adherence to the assigned intervention regimen that could have affected participants' outcomes?	PN	Long follow up period but most probably the majority stuck to the ttt regimen. Only 1 DC
	2.6. If N/PN/NI to 2.3, or Y/PY/NI to 2.4 or 2.5: Was an appropriate analysis used to estimate the effect of adhering to the intervention?	NA	
	**Risk of bias judgement**	**Low**	
Bias due to missing outcome data	3.1 Were data for this outcome available for all, or nearly all, participants randomized?	N	
	3.2 If N/PN/NI to 3.1: Is there evidence that result was not biased by missing outcome data?	PN	
	3.3 If N/PN to 3.2: Could missingness in the outcome depend on its true value?	N	
	3.4 If Y/PY/NI to 3.3: Is it likely that missingness in the outcome depended on its true value?	NA	
	**Risk of bias judgement**	**Low**	
Bias in measurement of the outcome	4.1 Was the method of measuring the outcome inappropriate?	N	
	4.2 Could measurement or ascertainment of the outcome have differed between intervention groups?	N	
	4.3 Were outcome assessors aware of the intervention received by study participants?	NI	
	4.4 If Y/PY/NI to 4.3: Could assessment of the outcome have been influenced by knowledge of intervention received?	N	Objective outcome
	4.5 If Y/PY/NI to 4.4: Is it likely that assessment of the outcome was influenced by knowledge of intervention received?	NA	
	**Risk of bias judgement**	**Low**	
Bias in selection of the reported result	5.1 Were the data that produced this result analysed in accordance with a pre-specified analysis plan that was finalized before unblinded	Y	Analysis plan was available before data analysis
	5.2 ... multiple eligible outcome measurements (e.g. scales, definitions, time points) within the outcome domain?	N	
	5.3 ... multiple eligible analyses of the data?	N	
	**Risk of bias judgement**	**Low**	
Overall bias	**Risk of bias judgement**	**Low**	

**Unique ID**	LVMI-Sardu	**Study ID**	Sardu 2021	**Assessor**	Ahmed
**Ref or Label**	6	**Aim**	adhering to intervention (the 'per-protocol' effect)	**The effect of adhering to intervention…**	occurance of non-protocol interventions; failures in implementing the intervention that could have affected the outcome; non-adherence to their assigned intervention by trial participants
**Experimental**	Metformin	**Comparator**	Placebo	**Source**	
**Outcome**	LVMI	**Results**		**Weight**	1

Domain	Signalling question	Response	Comments
**Bias arising from the randomization process**	1.1 Was the allocation sequence random?	PY	In the present study each patient received a unique sequential subject number by an Interactive Voice Response System (IVRS).
	1.2 Was the allocation sequence concealed until participants were enrolled and assigned to interventions?	PY	
	1.3 Did baseline differences between intervention groups suggest a problem with the randomization process?	N	
	**Risk of bias judgement**	**Low**	
**Bias due to deviations from intended interventions**	2.1 Were participants aware of their assigned intervention during the trial?	PN	Double blinded
	2.2 Were carers and people delivering the interventions aware of participants' assigned intervention during the trial?	PN	
	2.3. [If applicable:] If Y/PY/NI to 2.1 or 2.2: Were important non-protocol interventions balanced across intervention groups?	NA	
	2.4. [If applicable:] Were there failures in implementing the intervention that could have affected the outcome?	PN	
	2.5. [If applicable:] Was there non-adherence to the assigned intervention regimen that could have affected participants' outcomes?	PN	
	2.6. If N/PN/NI to 2.3, or Y/PY/NI to 2.4 or 2.5: Was an appropriate analysis used to estimate the effect of adhering to the intervention?	NA	
	**Risk of bias judgement**	**Low**	
**Bias due to missing outcome data**	3.1 Were data for this outcome available for all, or nearly all, participants randomized?	Y	All patients were analyzed
	3.2 If N/PN/NI to 3.1: Is there evidence that result was not biased by missing outcome data?	NA	
	3.3 If N/PN to 3.2: Could missingness in the outcome depend on its true value?	NA	
	3.4 If Y/PY/NI to 3.3: Is it likely that missingness in the outcome depended on its true value?	NA	
	**Risk of bias judgement**	**Low**	
**Bias in measurement of the outcome**	4.1 Was the method of measuring the outcome inappropriate?	N	
	4.2 Could measurement or ascertainment of the outcome have differed between intervention groups?	N	The examination was performed at baseline and after 12 months, according to the American Society of Echocardiography recommendations
	4.3 Were outcome assessors aware of the intervention received by study participants?	N	
	4.4 If Y/PY/NI to 4.3: Could assessment of the outcome have been influenced by knowledge of intervention received?	NA	
	4.5 If Y/PY/NI to 4.4: Is it likely that assessment of the outcome was influenced by knowledge of intervention received?	NA	
	**Risk of bias judgement**	**Low**	
**Bias in selection of the reported result**	5.1 Were the data that produced this result analysed in accordance with a pre-specified analysis plan that was finalized before unblinded outcome data were available for analysis?	PY	
	5.2 ... multiple eligible outcome measurements (e.g. scales, definitions, time points) within the outcome domain?	N	
	5.3 ... multiple eligible analyses of the data?	N	
	**Risk of bias judgement**	**Low**	
**Overall bias**	**Risk of bias judgement**	**Low**	

**Unique ID**	LVMI-Velázquez	**Study ID**	Velázquez 2015	**Assessor**	
**Ref or Label**	7	**Aim**	adhering to intervention (the 'per-protocol' effect)	**The effect of adhering to intervention…**	occurance of non-protocol interventions; failures in implementing the intervention that could have affected the outcome; non-adherence to their assigned intervention by trial participants
**Experimental**	Metformin	**Comparator**	SOC	**Source**	
**Outcome**	LVMI	**Results**		**Weight**	1

Domain	Signalling question	Response	Comments
**Bias arising from the randomization process**	1.1 Was the allocation sequence random?	PN	Not indicated
	1.2 Was the allocation sequence concealed until participants were enrolled and assigned to interventions?	NI	
	1.3 Did baseline differences between intervention groups suggest a problem with the randomization process?	PN	All were comparable
	**Risk of bias judgement**	**Some concerns**	
**Bias due to deviations from intended interventions**	2.1 Were participants aware of their assigned intervention during the trial?	Y	No placebo
	2.2 Were carers and people delivering the interventions aware of participants' assigned intervention during the trial?	Y	
	2.3. [If applicable:] If Y/PY/NI to 2.1 or 2.2: Were important non-protocol interventions balanced across intervention groups?	Y	
	2.4. [If applicable:] Were there failures in implementing the intervention that could have affected the outcome?	PN	
	2.5. [If applicable:] Was there non-adherence to the assigned intervention regimen that could have affected participants' outcomes?	PN	
	2.6. If N/PN/NI to 2.3, or Y/PY/NI to 2.4 or 2.5: Was an appropriate analysis used to estimate the effect of adhering to the intervention?	NA	
	**Risk of bias judgement**	**Low**	
**Bias due to missing outcome data**	3.1 Were data for this outcome available for all, or nearly all, participants randomized?	Y	
	3.2 If N/PN/NI to 3.1: Is there evidence that result was not biased by missing outcome data?	NA	
	3.3 If N/PN to 3.2: Could missingness in the outcome depend on its true value?	NA	
	3.4 If Y/PY/NI to 3.3: Is it likely that missingness in the outcome depended on its true value?	NA	
	**Risk of bias judgement**	**Low**	
**Bias in measurement of the outcome**	4.1 Was the method of measuring the outcome inappropriate?	N	
	4.2 Could measurement or ascertainment of the outcome have differed between intervention groups?	N	No, objective measurment
	4.3 Were outcome assessors aware of the intervention received by study participants?	PY	
	4.4 If Y/PY/NI to 4.3: Could assessment of the outcome have been influenced by knowledge of intervention received?	N	
	4.5 If Y/PY/NI to 4.4: Is it likely that assessment of the outcome was influenced by knowledge of intervention received?	NA	
	**Risk of bias judgement**	**Low**	
**Bias in selection of the reported result**	5.1 Were the data that produced this result analysed in accordance with a pre-specified analysis plan that was finalized before unblinded outcome data were available for analysis?	N	No specified protocol. However, no direct comparison between groups
	5.2 ... multiple eligible outcome measurements (e.g. scales, definitions, time points) within the outcome domain?	N	
	5.3 ... multiple eligible analyses of the data?	N	
	**Risk of bias judgement**	**Some concerns**	
**Overall bias**	**Risk of bias judgement**	**Some concerns**	

Unique ID	LVMI-Ali	Study ID	Ali 2016	Assessor	Ahmed
Ref or Label	8	Aim	adhering to intervention (the 'per-protocol' effect)	The effect of adhering to intervention…	occurance of non-protocol interventions; failures in implementing the intervention that could have affected the outcome; non-adherence to their assigned intervention by trial participants
Experimental	Metformin	Comparator	Placebo	Source	Journal article(s)
Outcome	LVMI	Results		Weight	1

Domain	Signalling question	Response	Comments
Bias arising from the randomization process	1.1 Was the allocation sequence random?	Y	Block randomization. Manufacturing and packaging including blinding was performed by Stichting Apotheek Haagse Ziekenhuizen, Den
	1.2 Was the allocation sequence concealed until participants were enrolled and assigned to interventions?	Y	
	1.3 Did baseline differences between intervention groups suggest a problem with the randomization process?	N	
	**Risk of bias judgement**	**Low**	
Bias due to deviations from intended interventions	2.1 Were participants aware of their assigned intervention during the trial?	PN	Double blinded randomized RCT
	2.2 Were carers and people delivering the interventions aware of participants' assigned intervention during the trial?	PN	
	2.3. [If applicable:] If Y/PY/NI to 2.1 or 2.2: Were important non-protocol interventions balanced across intervention groups?	NA	
	2.4. [If applicable:] Were there failures in implementing the intervention that could have affected the outcome?	PN	
	2.5. [If applicable:] Was there non-adherence to the assigned intervention regimen that could have affected participants' outcomes?	PN	
	2.6. If N/PN/NI to 2.3, or Y/PY/NI to 2.4 or 2.5: Was an appropriate analysis used to estimate the effect of adhering to the intervention?	NA	
	**Risk of bias judgement**	**Low**	
Bias due to missing outcome data	3.1 Were data for this outcome available for all, or nearly all, participants randomized?	N	A total of 380 patients were randomized. However, only ~140 completed the study.
	3.2 If N/PN/NI to 3.1: Is there evidence that result was not biased by missing outcome data?	PN	LVEF values were imputed in the original study and results did not differ
	3.3 If N/PN to 3.2: Could missingness in the outcome depend on its true value?	PN	Double blinded and missing proportions were similar across groups. Reasons for missingness were also indicated
	3.4 If Y/PY/NI to 3.3: Is it likely that missingness in the outcome depended on its true value?	NA	
	**Risk of bias judgement**	**Low**	
Bias in measurement of the outcome	4.1 Was the method of measuring the outcome inappropriate?	N	Echocardiography
	4.2 Could measurement or ascertainment of the outcome have differed between intervention groups?	N	
	4.3 Were outcome assessors aware of the intervention received by study participants?	PN	Double blinding but unblinding date not stated
	4.4 If Y/PY/NI to 4.3: Could assessment of the outcome have been influenced by knowledge of intervention received?	NA	
	4.5 If Y/PY/NI to 4.4: Is it likely that assessment of the outcome was influenced by knowledge of intervention received?	NA	
	**Risk of bias judgement**	**Low**	
Bias in selection of the reported result	5.1 Were the data that produced this result analysed in accordance with a pre-specified analysis plan that was finalized before unblinded outcome data were available for analysis?	Y	
	5.2 ... multiple eligible outcome measurements (e.g. scales, definitions, time points) within the outcome domain?	N	
	5.3 ... multiple eligible analyses of the data?	N	
	**Risk of bias judgement**	**Low**	
**Overall bias**	**Risk of bias judgement**	**Low**	
